# Thermal-History-Mediated Defect–Microstructure–Property Evolution in Multi-Laser Powder Bed Fusion of TC11 Titanium Alloy Structures

**DOI:** 10.3390/ma19183956

**Published:** 2026-09-17

**Authors:** Liubaixiang He, Junfeng He, Jiamin Zhang, Xin Lin, Xufei Lu

**Affiliations:** 1 State Key Laboratory of Solidification Processing, Northwestern Polytechnical University, Xi’an 710072, China; 2 MIIT Key Laboratory of Metal High Performance Additive Manufacturing and Innovative Design, Northwestern Polytechnical University, Xi’an 710072, China; 3 International Centre for Numerical Methods in Engineering, Universidad Politécnica de Cataluña, 08034 Barcelona, Spain; jiamin.zhang@upc.edu

**Keywords:** multi-laser powder bed fusion, TC11 titanium alloy, inter-laser overlap zone, process–defect–microstructure–property relationship, fracture behavior

## Abstract

Multi-laser powder bed fusion (PBF-ML) enables efficient fabrication of large components but introduces spatially heterogeneous thermal histories near inter-laser overlap zones. However, how global thermal-input variables and local thermal-path variations jointly affect defect evolution, microstructure, and mechanical response in geometrically constrained Ti-6.5Al-3.5Mo-1.5Zr-0.3Si (TC11) structures remains unclear. Here, TC11 arch structures were fabricated by varying laser power, scanning speed, overlap-zone position, and interlayer rotation and characterized by tensile testing, optical metallography, Scanning Electron Microscope (SEM), and fractography. Increasing laser power from 170 to 290 W increased Yield Strength (YS) and Ultimate Tensile Strength (UTS) from 955 and 1228 MPa to 1259 and 1463 MPa, respectively, while Elongation (EL) decreased from 14.7% to 3.1%. Increasing the scanning speed from 1050 to 1450 mm/s produced the opposite strength–ductility trend, whereas overlap-zone position had limited influence on strength and 67° interlayer rotation produced moderate improvements. Defect count and area fraction did not always vary synchronously, while semi-quantitative SEM analysis revealed microstructural coarsening with increasing laser power and refinement with increasing scanning speed. These results show that global thermal-input variables primarily govern the overall strength–ductility and microstructural-scale responses, whereas local thermal-path variables mainly regulate spatial defects and microstructural heterogeneity.

## 1. Introduction

Laser powder bed fusion (PBF-L) has become an important additive manufacturing technology for fabricating metallic components with complex geometries due to its high design flexibility and near-net-shape capability [1,2,3]. During PBF-L processing, repeated cycles of localized laser heating, rapid solidification, and subsequent layer-wise thermal cycling generate highly transient thermal histories. These thermal histories govern phase transformation, microstructural evolution, residual stress development, and the resulting mechanical behavior of additively manufactured titanium alloys [4,5,6]. In addition, process-induced defects, including lack-of-fusion defects and keyhole-related pores, can act as preferential sites for strain localization and crack initiation, leading to variations in mechanical performance [7,8,9]. These coupled effects are particularly important for titanium alloys because their acicular and lamellar transformation structures are highly sensitive to cooling conditions and repeated thermal exposure.

Multi-laser powder bed fusion (PBF-ML) has been developed to improve manufacturing efficiency and extend the applicability of powder bed fusion technology to large-scale metallic components. Compared with conventional PBF-L systems, PBF-ML divides the build area into multiple laser fields, where independent lasers simultaneously process assigned regions. However, laser-field partitioning inevitably introduces inter-laser overlap zones, where scan-vector allocation, heat-flow paths, thermal accumulation, and layer-wise reheating behavior may differ from those in individual laser fields [10,11,12,13]. As a result, the overlap zone may exhibit distinct defect characteristics and microstructural features compared with surrounding regions. Under appropriate laser-field coordination, sufficient metallurgical continuity can be maintained, whereas inadequate coordination may lead to localized defects, surface irregularities, and microstructural heterogeneity. Therefore, the inter-laser overlap zone represents a characteristic feature of PBF-ML and requires separate consideration from conventional PBF-L processing.

TC11 is an α + β heat-resistant titanium alloy widely used in aerospace applications because of its high specific strength, thermal stability, and elevated-temperature capability [14,15,16]. Previous studies have shown that the rapid solidification and repeated thermal cycling inherent to PBF-L promote the formation of acicular α′ and related acicular–lamellar morphologies in TC11, while processing parameters and local reheating further influence prior-β grain characteristics, lath morphology, phase distribution, residual stress, and microstructural uniformity [14,15,16,17,18,19]. In particular, the locally modified thermal history associated with multiple laser fields may further influence the spatial arrangement and deformation accommodation capability of acicular–lamellar structures. However, compared with the extensively studied Ti-6Al-4V alloy, the process–microstructure–property relationships of PBF-ML-fabricated TC11 remain insufficiently understood.

For conventional PBF-L processing, extensive studies have investigated the effects of processing parameters on defect formation, microstructure evolution, and mechanical properties of titanium alloys. Laser power and scanning speed determine melt-pool stability, thermal exposure, and solidification conditions, thereby influencing lack-of-fusion defects, keyhole-related defects, transformed microstructure, and strength–ductility balance [7,16,17,20,21]. Meanwhile, scanning strategies, including stripe scanning, island scanning, and interlayer rotation, modify heat accumulation, thermal cycling paths, residual stress evolution, texture characteristics, and mechanical response by changing the spatial distribution of laser trajectories [18,19,22,23]. Related directed energy deposition (DED) studies have further demonstrated that variations in thermal history can strongly influence grain morphology, layer-band formation, residual stress, and deformation behavior in titanium alloys [24,25,26]. These studies provide a fundamental understanding of thermal-history-dependent microstructure and property evolution in laser-based additive manufacturing.

However, the relationships established for single-laser PBF-L cannot be directly transferred to PBF-ML because multiple laser fields introduce additional spatial and temporal variations in thermal history. In PBF-ML, laser power and scanning speed determine the overall thermal input and melt-pool behavior, whereas laser-field allocation, overlap-zone position, and interlayer rotation influence local heat-flow paths and thermal-cycle distribution near field boundaries [11,12,13,27]. In addition, the coordination between adjacent lasers may affect melt-pool interactions, vapor plume behavior, and defect formation processes [11,28]. These locally heterogeneous thermal conditions can modify microstructural morphology, defect distribution, and deformation behavior between overlap and non-overlap regions [28,29]. Therefore, understanding the influence of laser-field coordination and scanning strategies on local thermal history is essential for evaluating the mechanical response of PBF-ML components.

Previous studies on PBF-ML have mainly focused on the consistency between inter-laser overlap zones and single-laser regions. Li et al. [30] reported that optimization of hatch–angle relationships reduced surface irregularities caused by repeated laser exposure and improved the consistency of microstructure and tensile properties across overlap regions. Wei et al. [13] investigated overlap-zone characteristics in Ti6Al4V and demonstrated that defect formation and mechanical response were strongly influenced by laser-field coordination. For the α + β/near-α TA15 alloy, Li et al. [28] observed that laser switching-related overlap regions exhibited directionally distributed pores, while increased thermal accumulation caused by multiple laser interactions promoted acicular structure coarsening and reduced microhardness. Numerical studies by Zhang et al. [27] further showed that scan direction, interlayer rotation, and temporal delay influenced temperature evolution, residual stress, and deformation behavior during multi-laser processing. Other investigations have also demonstrated that overlap width and repeated exposure conditions affect microstructural consistency and mechanical uniformity between overlap and non-overlap regions [29,31]. These studies indicate that overlap-zone behavior is controlled by the combined effects of laser-field coordination, thermal accumulation, and local defect evolution.

The mechanical response of PBF-ML titanium alloys is determined not only by defect quantity but also by defect morphology, spatial distribution, and the deformation accommodation capability of the transformed microstructure. Irregular lack-of-fusion defects generally introduce stronger stress concentrations than relatively spherical pores, while microstructural characteristics such as lath scale, packet arrangement, and local heterogeneity influence strain accommodation and crack propagation behavior [8,9,32,33]. Therefore, the tensile behavior of PBF-ML components should be interpreted by considering the coupled effects of defect characteristics, microstructural architecture, fracture behavior, and thermal history rather than relying solely on defect frequency or relative density.

Although PBF-ML provides an effective approach for improving the productivity of large and complex components, most previous studies have been performed using simple bulk coupons or idealized overlap configurations [12,13,27,29,30,31]. Such specimens cannot fully represent the geometry-dependent heat dissipation, local thermal accumulation, scan-vector variation, and structural constraint conditions encountered in practical components. Consequently, how inter-laser overlap zones interact with geometrically constrained structures, and how this interaction affects defect evolution, microstructural heterogeneity, and mechanical response, remains insufficiently resolved.

Different from previous PBF-ML studies based mainly on bulk coupons or idealized overlap configurations, the present work employs a geometrically constrained arch structure to distinguish global thermal-input effects, controlled primarily by laser power and scanning speed, from local thermal-path effects associated with overlap-zone position and interlayer rotation. By directly correlating overlap/non-overlap defects and microstructural heterogeneity with tensile and fracture behaviors, this study provides new insight into the thermal-history-dependent mechanical response of geometrically constrained PBF-ML TC11 structures.

## 2. Experimental Methods

### 2.1. Materials and Process Parameters

**Figure 1:**
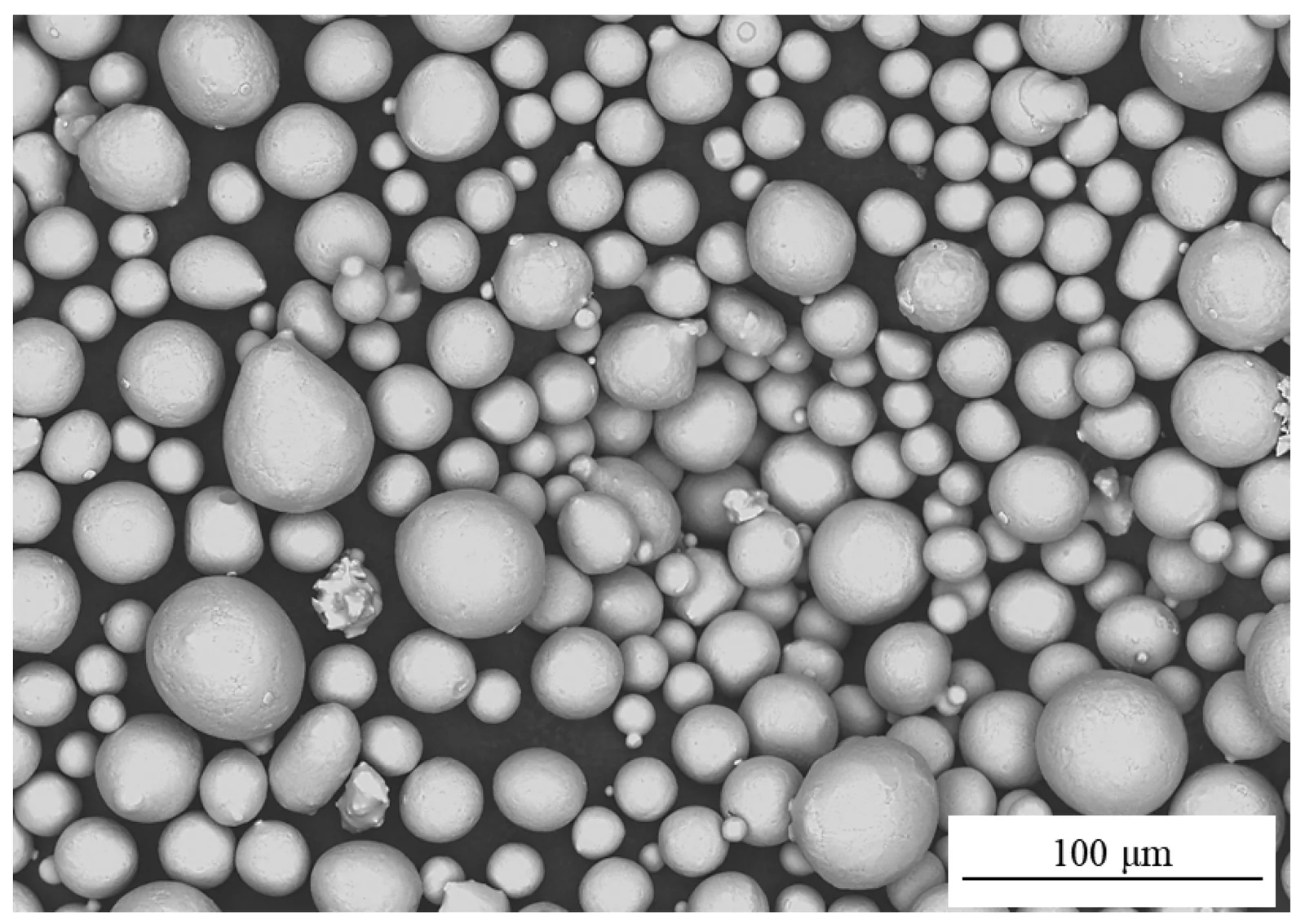
SEM morphology of the gas-atomized TC11 titanium-alloy powder.

Gas-atomized TC11 titanium-alloy powder produced by electrode induction melting inert gas atomization (EIGA) was used as the feedstock material. The particle-size distribution ranged from 15 to 53 μm. The powder morphology is shown in Figure 1, and the nominal chemical composition is listed in Table 1. Before fabrication, the powder was vacuum dried at 130 °C for 5 h to minimize moisture adsorption during storage and handling.

All specimens were fabricated on a Ti6Al4V substrate with dimensions of 400 mm × 250 mm × 50 mm. Before fabrication, the substrate surface was mechanically ground and ultrasonically cleaned in acetone to remove surface contaminants.

Fabrication was performed using a BLT-S400 PBF-ML system (Xi’an Bright Laser Technologies Co., Ltd., Xi’an, China) equipped with three fiber lasers operating at wavelengths of 1060−1080 nm. The system supports layer thicknesses of 20−100 μm and a maximum scanning speed of 7 m/s. No substrate preheating was applied during fabrication. This condition was intentionally maintained to provide a consistent thermal boundary condition and to isolate the effects of laser power, scanning speed, overlap-zone position, and interlayer rotation without introducing an additional global thermal variable. High-purity argon was continuously supplied throughout the building process, and the chamber oxygen concentration was maintained below 100 ppm.

A single-factor experimental design was employed to investigate four processing variables, including laser power, scanning speed, inter-laser overlap-zone position, and interlayer-rotation strategy. These variables were selected to represent different aspects of thermal-history regulation during PBF-ML processing. Laser power and scanning speed primarily modify the overall thermal input, whereas overlap-zone position and interlayer rotation influence the spatial distribution and directional characteristics of layer-wise thermal cycling. Except for the investigated variable, all other processing parameters were kept constant.

The reference processing condition consisted of a laser power of 230 W, a scanning speed of 1250 mm/s, a layer thickness of 40 μm, a hatch spacing of 0.11 mm, and a laser spot diameter of 0.10 mm. Stripe scanning was adopted with a stripe width of 5 mm, an inter-stripe overlap of 0.05 mm, and a 67° rotation between successive layers.

During fabrication, the three lasers operated simultaneously, with each laser processing the scan vectors assigned to its corresponding laser field. The nominal width of the inter-laser overlap zone was 5 mm. In this study, the inter-laser overlap zone refers to the boundary region where adjacent laser fields were connected. No additional sequential exposure or intentional remelting operation was applied in this region. Therefore, the overlap-zone characteristics were determined by the coordination between neighboring laser fields and the associated local thermal-history variation.

The specimen fabricated under the reference condition was used as the common reference for all parameter series, corresponding to P0 for laser power, V0 for scanning speed, A1 for overlap offset, and R67 for interlayer rotation. In the laser-power series, P1–P4 denote specimens fabricated with different laser powers while the other processing parameters were kept at the reference values. Similarly, V1–V4 represent specimens with varied scanning speeds, and A1–A3 represent different overlap offsets. For the interlayer-rotation series, R0 denotes the specimen fabricated without interlayer rotation (0°), whereas R67 represents the reference condition with a 67° interlayer rotation. The specific parameter levels for each series are summarized in Table 2.

### 2.2. Arch Geometry and Layout

An arch-shaped specimen containing a central semicircular opening was designed as a geometrically constrained model structure to investigate the interaction between inter-laser overlap zones and structural-induced thermal variations during PBF-ML processing. Unlike conventional bulk coupons with relatively uniform heat dissipation conditions, the arch geometry introduces spatial variations in heat-transfer paths and local constraint conditions, providing a controlled configuration for evaluating the influence of multi-laser thermal heterogeneity on mechanical response.

**Figure 2:**
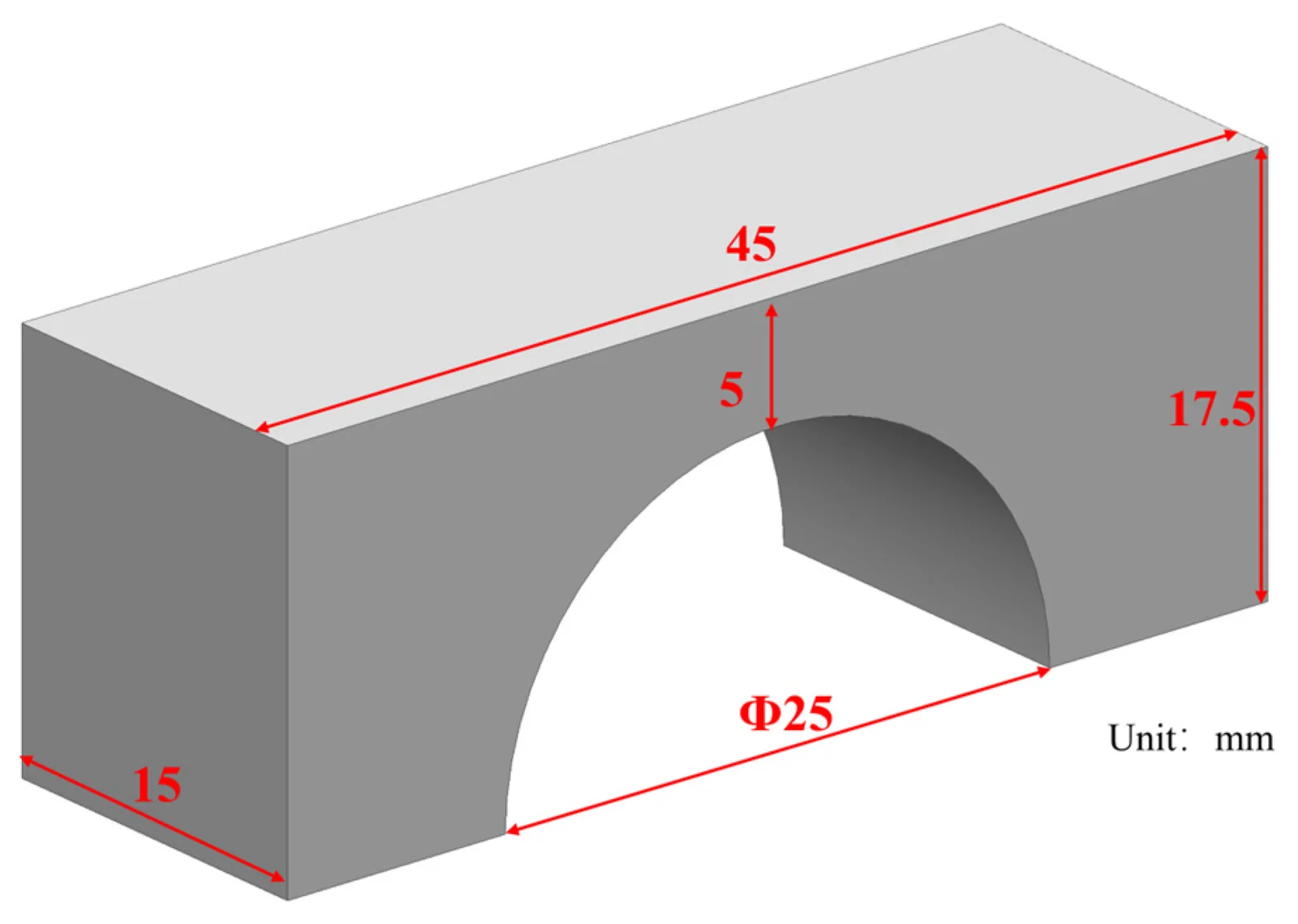
Geometry and dimensions of the TC11 arch specimen (unit: mm).

As illustrated in Figure 2, the specimen had a length of 45 mm, a width of 15 mm, and a height of 17.5 mm. The central semicircular opening had a diameter of 25 mm, resulting in a minimum ligament thickness of 5 mm between the crown of the opening and the upper surface. All specimens were fabricated using identical dimensions and build orientations to maintain consistent geometric and thermal boundary conditions among different processing conditions.

**Figure 3:**
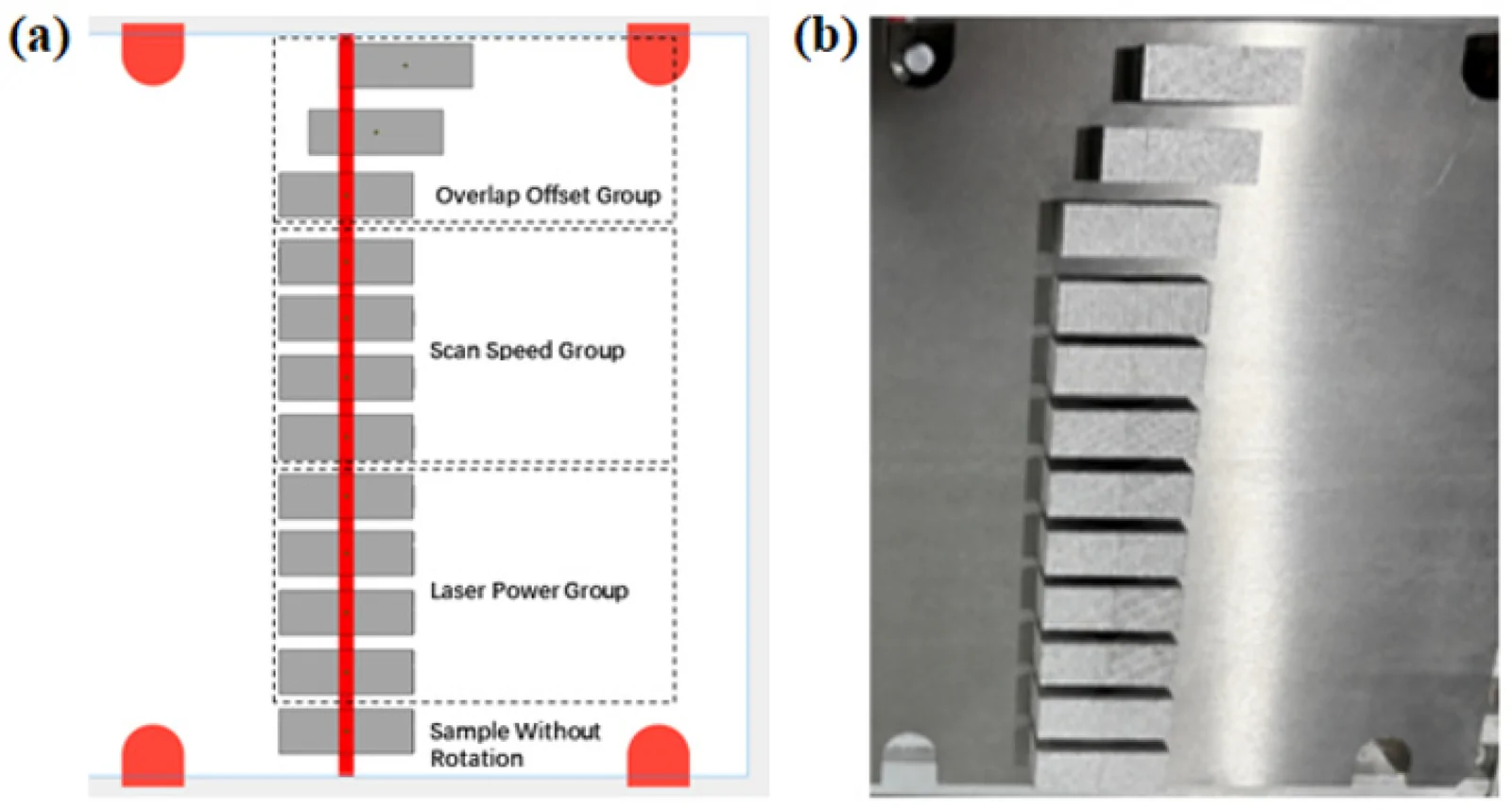
Build-platform layout and representative as-built specimen arrangement: (**a**) schematic distribution of the different processing groups, where the red vertical band denotes the inter-laser overlap zone; (**b**) corresponding as-built TC11 arch specimens.

As shown in Figure 3a, the red vertical band denotes the inter-laser overlap zone, i.e., the boundary region shared by adjacent laser fields and the red shapes at the four corners of the substrate denote the screw mounting areas for substrate fixation. Regions away from this red-marked zone are defined as non-overlap regions and are primarily processed within a single laser field. Accordingly, the overlap and non-overlap regions used in the subsequent defect and microstructural characterizations refer to observation locations inside and outside the red-marked overlap zone, respectively. The term “overlap offset” in Table 2 refers to the relative longitudinal position of this inter-laser overlap zone with respect to the arch specimen geometry. The corresponding as-printed physical specimens are shown in Figure 3b.

For the laser-power, scanning-speed, and interlayer-rotation series, the centerline of the nominal 5 mm inter-laser overlap zone was aligned with the longitudinal centerline of the arch specimen. This configuration ensured that the overlap zone was located within the tensile sampling region while maintaining identical geometric conditions among different processing variables.

A separate overlap-zone-position series was designed to investigate the influence of the relative location between the inter-laser overlap zone and the arch geometry. In this series, the overlap-zone centerline was positioned at the arch center or shifted longitudinally by 10 and 20 mm, while the specimen geometry and all other processing parameters remained unchanged.

In this study, the inter-laser overlap-zone position refers to the location of the boundary region where adjacent laser fields are connected. It is distinct from the 0.05 mm overlap between neighboring scan stripes used in stripe scanning. The former represents a PBF-ML field-allocation characteristic, whereas the latter is an intrinsic parameter of the scanning strategy.

**Figure 4:**
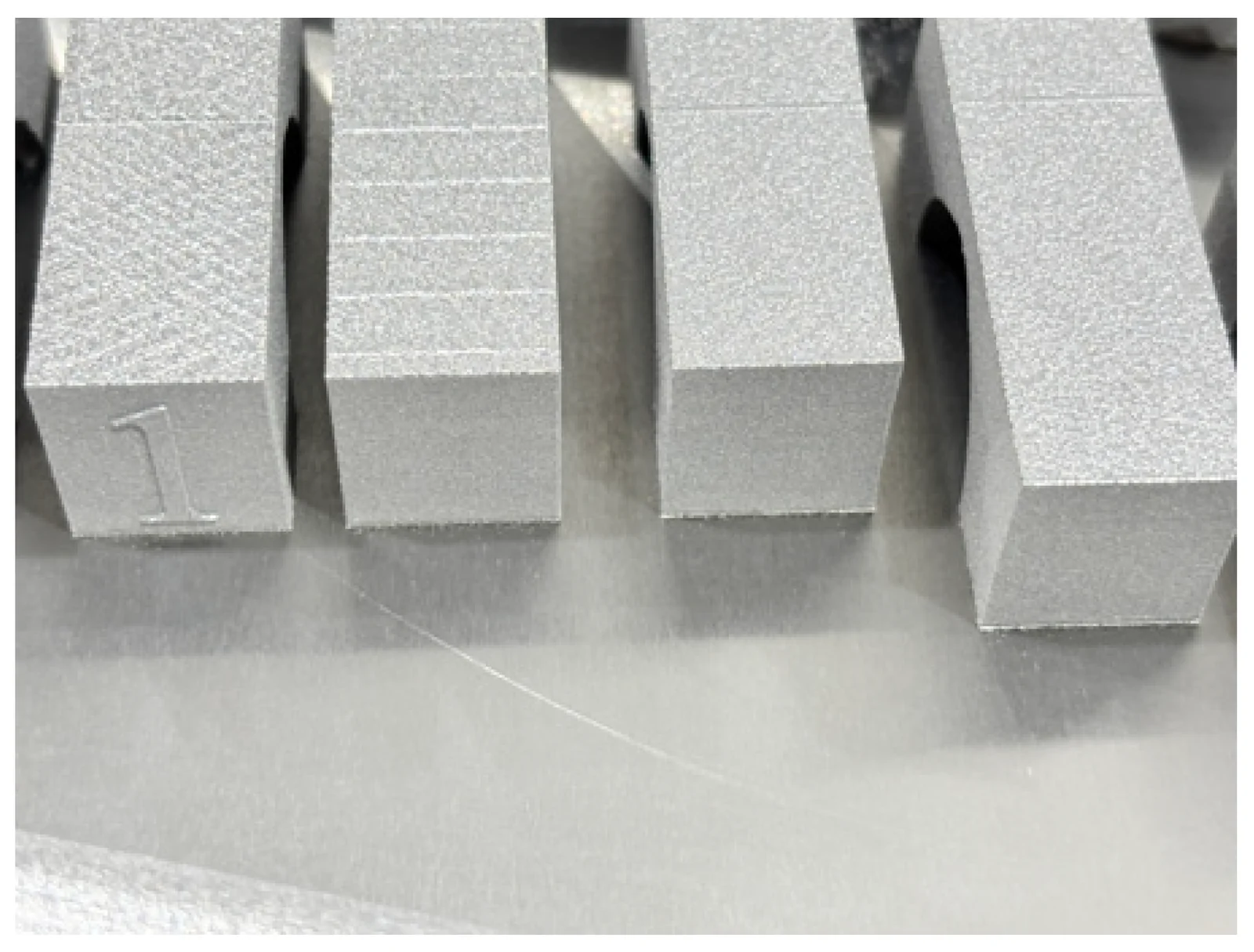
Fabrication and arrangement of the PBF-ML TC11 as-built specimens.

The actual build arrangement and as-fabricated arch specimens are presented in Figure 4.

### 2.3. Specimen Preparation and Characterization

**Figure 5:**
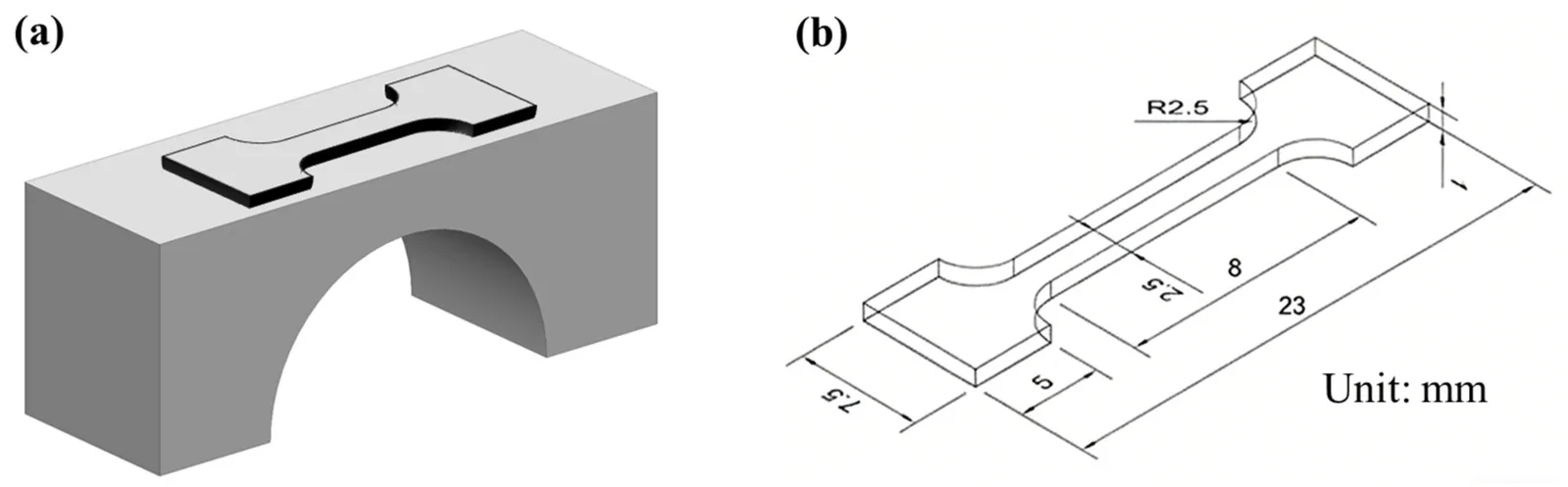
Sampling scheme and geometry of the miniature tensile specimen: (**a**) extraction locations of tensile and metallographic specimens and (**b**) tensile-specimen dimensions (unit: mm).

Miniature flat tensile specimens were extracted from the upper solid region of the arch specimens by wire electrical discharge machining (WEDM), as illustrated in Figure 5a. The tensile axis was parallel to the longitudinal direction of the arch structure. As shown in Figure 5b, the tensile specimens had an overall length of 23 mm, a gauge length of 8 mm, a gauge width of 2.5 mm, a grip width of 7.5 mm, a transition radius of 2.5 mm, and a nominal thickness of 1 mm. The actual width and thickness of each specimen were measured before testing and used to calculate the initial cross-sectional area. The miniature tensile geometry enabled mechanical characterization of the constrained arch structures while maintaining consistent sampling conditions among different processing states.

Room-temperature tensile tests were performed using a Shimadzu AGS-X universal testing machine (Shimadzu Corporation, Kyoto, Japan) equipped with a 10 kN load cell. Axial strain was measured using a clip-on extensometer with a gauge length of 7 mm. The tests were conducted at a nominal strain rate of 1.0 × 10^−3^ s^−1^ and continued until fracture. Room-temperature tensile testing followed the general principles of ASTM E8/E8M [34] and ISO 6892-1 [35], while a miniaturized specimen geometry was adopted because of the dimensional constraint of the arch structure. The 0.2% proof strength (YS), ultimate tensile strength (UTS), and elongation (EL) were obtained from the engineering stress–strain curves. After tensile testing, fracture surfaces were examined by Verios G4 SEM (Thermo Fisher Scientific, Hillsboro, OR, USA) to characterize fracture features and their relationship with processing conditions and tensile response. One tensile specimen was tested for each processing condition; therefore, the reported tensile values are interpreted as condition-specific responses rather than statistically averaged properties.

For defect and microstructural characterization, two rectangular specimens with dimensions of 10 mm × 5 mm × 10 mm were extracted by WEDM from regions adjacent to the tensile sampling locations. Each metallographic specimen was positioned 2 mm from the outer end of the tensile gauge section to maintain spatial correspondence between the observed microstructural/defect characteristics and the corresponding mechanical response.

For defect characterization, the sectioned specimens were hot-mounted in resin at 150 °C under a pressure of 22 MPa for 4 min and subsequently cooled to 40 °C before removal. The exposed surfaces were sequentially ground using 240-, 600-, 1000-, 1500-, 2000-, 4000-, and 7000-grit SiC papers, followed by polishing with 0.05 μm OPS colloidal silica suspension. The polished unetched surfaces were examined by optical microscopy.

For each processing condition, two equal-area fields at identical magnification were selected using a consistent image-selection criterion. The observed dark defect features were converted into binary maps for visualization and quantitative analysis. The number of discrete defect features was counted separately in each field, and the average value was used as an indicator of apparent two-dimensional defect frequency. In addition, the projected areas of all segmented defect features were summed and normalized by the total observation area to obtain the apparent two-dimensional defect area fraction. Thus, the defect count characterizes the occurrence frequency of discrete defects, whereas the area fraction provides an additional size-sensitive measure of the overall defect occupancy. Because both parameters are obtained from two-dimensional metallographic sections, they do not directly represent three-dimensional defect volume or connectivity and were therefore interpreted together with the observed defect morphology and spatial distribution.

For microstructural characterization, the polished specimens were etched using Kroll’s reagent (2 mL HF, 4 mL HNO_3_, and 100 mL distilled water), rinsed with distilled water, cleaned with ethanol, and dried. The etched surfaces were examined by SEM to characterize the acicular–lamellar morphology and microstructural differences between the inter-laser overlap zone and non-overlap regions. To provide a semi-quantitative comparison of the microstructural scale, the apparent width of clearly distinguishable acicular features was measured from SEM micrographs using the image scale bars. Measurements were taken approximately normal to the longitudinal direction of the acicular features, and the observed width range was recorded for each processing condition. Because the individual α/α′ phases cannot be unambiguously distinguished from the present etched SEM observations, the measured values are reported as approximate apparent acicular-feature widths rather than phase-specific lath thicknesses.

## 3. Results and Discussion

The tensile response of PBF-ML titanium alloys is inherently governed by multiple coupled factors, including defect characteristics, transformed microstructure, residual stress, crystallographic texture, and their spatial heterogeneity. In the present study, the mechanistic interpretation focuses primarily on the experimentally characterized defect state, acicular–lamellar morphology, and fracture behavior. Residual stress and crystallographic texture, which are known to contribute to the mechanical response of additively manufactured titanium alloys, are considered additional possible factors but were not independently quantified here. Thermal-history variations discussed below are inferred from the controlled processing parameters and corresponding material responses rather than from direct in situ temperature measurements. Similarly, the defect statistics discussed below should be interpreted as apparent two-dimensional descriptors derived from metallographic sections rather than direct measures of the three-dimensional defect population. Three-dimensional characterization techniques, such as X-ray computed tomography, would provide a more comprehensive assessment of defect volume, spatial distribution, and connectivity.

### 3.1. Effect of Laser Power on Defect–Microstructure Evolution and Tensile Response

**Figure 6:**
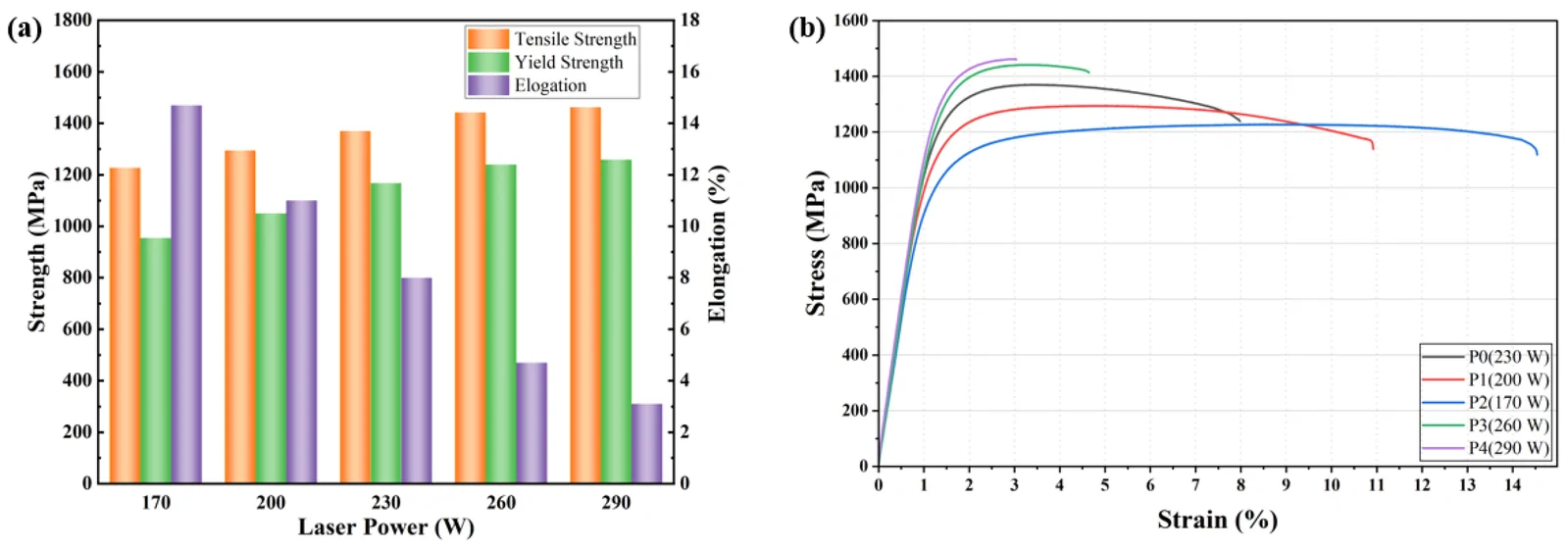
Tensile properties of specimens fabricated with different laser powers: (**a**) strength and elongation values and (**b**) engineering stress–strain curves.

The influence of laser power on the tensile response of multi-laser fabricated TC11 arch structures is shown in Figure 6. Increasing the laser power from 170 to 290 W increased the 0.2% proof strength (YS) from 955 to 1259 MPa and the ultimate tensile strength (UTS) from 1228 to 1463 MPa, whereas the elongation (EL) decreased continuously from 14.7% to 3.1%. The engineering stress–strain curves exhibited a gradual increase in flow stress and a reduction in plastic deformation accommodation capability with increasing laser power. These results indicate that laser power is a dominant parameter governing the strength–ductility balance by modifying the thermal conditions during melting and subsequent thermal cycling.

At relatively low laser power, insufficient thermal input limits melt-pool dimensions and lifetime, which may hinder complete powder melting and interlayer bonding. Increasing the laser power promotes more complete melting and reduces incomplete-fusion-related discontinuities, thereby increasing the effective load-bearing capability. However, excessive laser power leads to increased thermal accumulation and prolonged high-temperature exposure during layer deposition. The resulting thermal conditions may promote melt-pool instability and microstructural coarsening, causing a significant reduction in plastic deformation accommodation capability despite the increase in strength. To clarify the origin of this strength–ductility variation, the corresponding fracture features, defect characteristics, and microstructural responses are subsequently examined.

**Figure 7:**
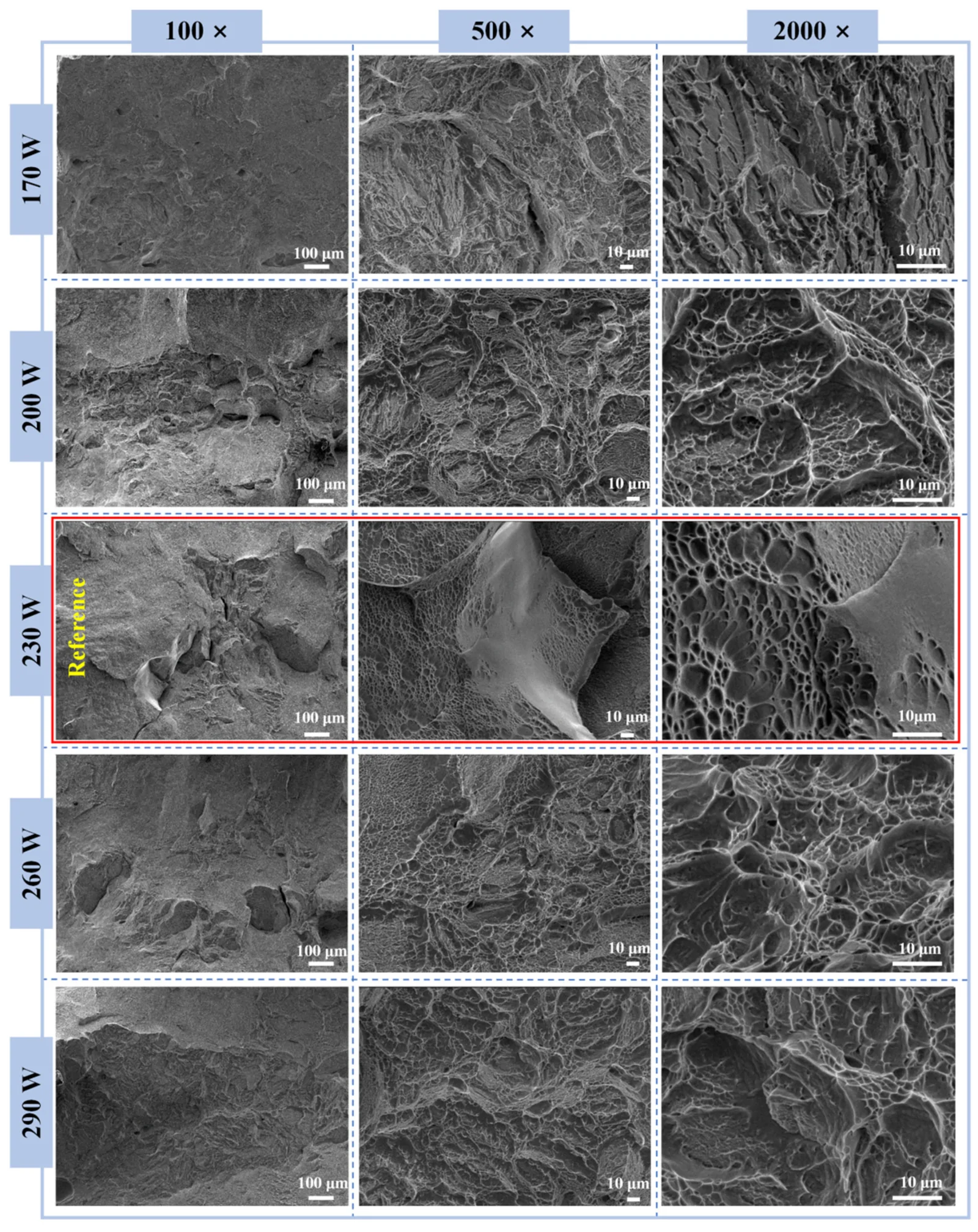
Fracture morphologies of specimens fabricated with different laser powers. The columns correspond to magnifications of 100×, 500×, and 2000× from left to right. And the red box indicates the reference group.

The fracture morphologies of specimens fabricated with different laser powers are presented in Figure 7. At 100× magnification, the low- and intermediate-power specimens exhibited relatively uniform overall fracture surfaces, whereas increasing laser power resulted in more heterogeneous macroscopic fracture features, particularly at 290 W. At 500× magnification, localized cavity-like regions and tearing features became more evident with increasing laser power. At 2000× magnification, microvoid-coalescence features and dimples were clearly observed under all conditions, while the high-power specimens exhibited a less uniform dimple morphology together with locally flatter regions. These observations are consistent with the pronounced decrease in elongation at high laser power.

**Figure 8:**
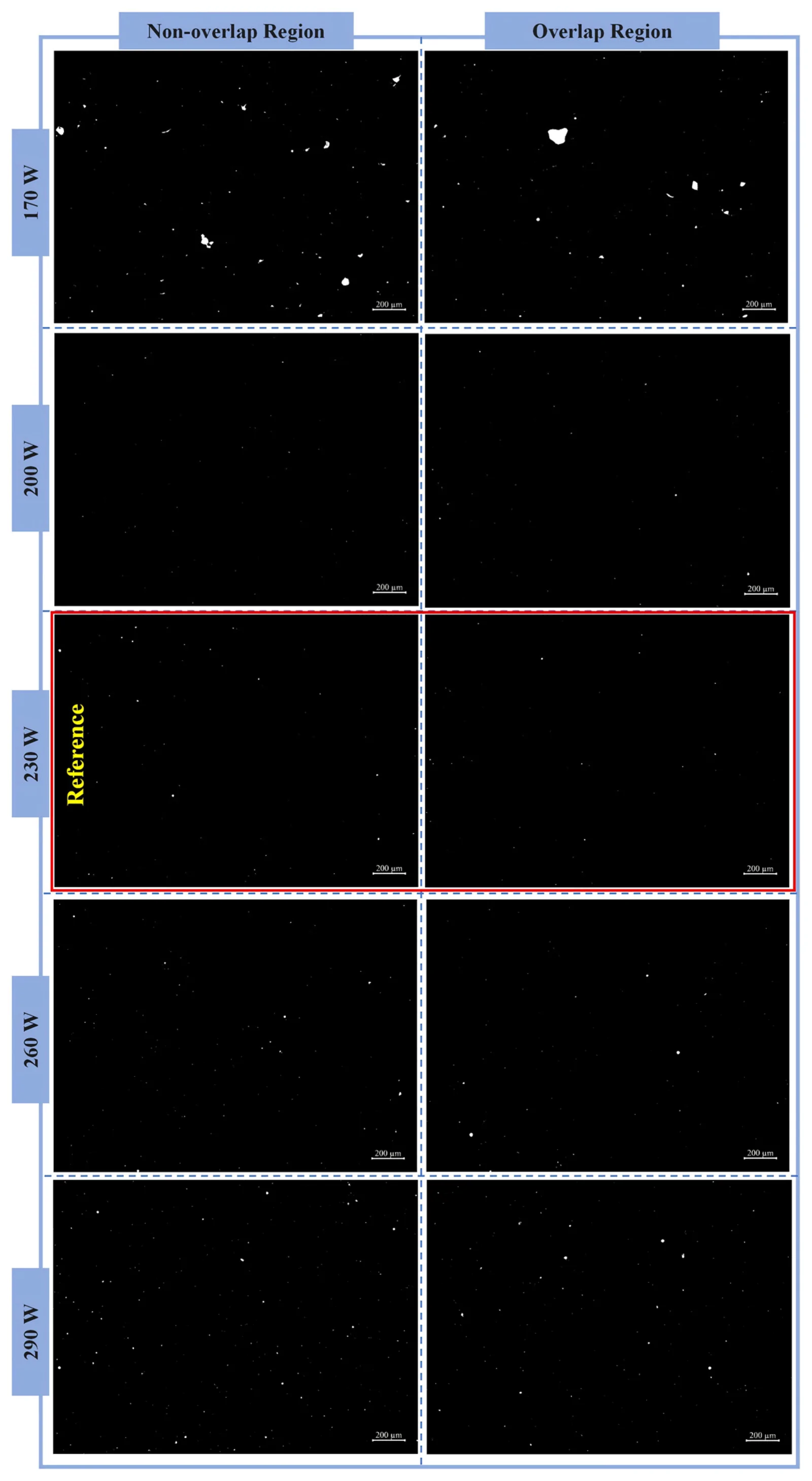
Binary defect maps obtained from polished cross-sections of specimens fabricated with different laser powers. And the red box indicates the reference group.

The binary defect maps obtained from polished cross-sections are shown in Figure 8, and the corresponding apparent two-dimensional defect statistics are summarized in Table 3. The average defect count decreased from 229 at 170 W to a minimum of 91 at 230 W and subsequently increased to 383 at 290 W. In contrast, the mean defect area fraction decreased from approximately 0.500% at 170 W to a minimum of approximately 0.110% at 200 W and then gradually increased with further increases in laser power. The different trends in defect count and area fraction indicate that defect frequency and characteristic defect size evolved simultaneously with laser power. Therefore, the defect state cannot be adequately described by defect count alone.

At low laser power, insufficient thermal input can limit melt-pool dimensions and lifetime, increasing the probability of relatively large incomplete-fusion-related discontinuities. Increasing laser power improves melting and fusion continuity, whereas excessive thermal exposure may increase melt-pool instability and the number of irregular defects. The combined evolution of defect count and area fraction therefore reflects a transition from insufficient fusion at low thermal input to increasingly unstable melting at high thermal input.

The defect distributions between the inter-laser overlap zone and non-overlap region are further compared in Figure 8. Within the examined fields, the overlap zone generally exhibited a lower apparent defect count than the corresponding non-overlap region. However, the defect area fraction did not show the same consistent regional dependence. This result indicates that the inter-laser overlap primarily modified the number and characteristic size distribution of detectable defects rather than uniformly reducing the overall defect content. Therefore, the regional defect state should be evaluated by considering both defect count and area fraction.

**Figure 9:**
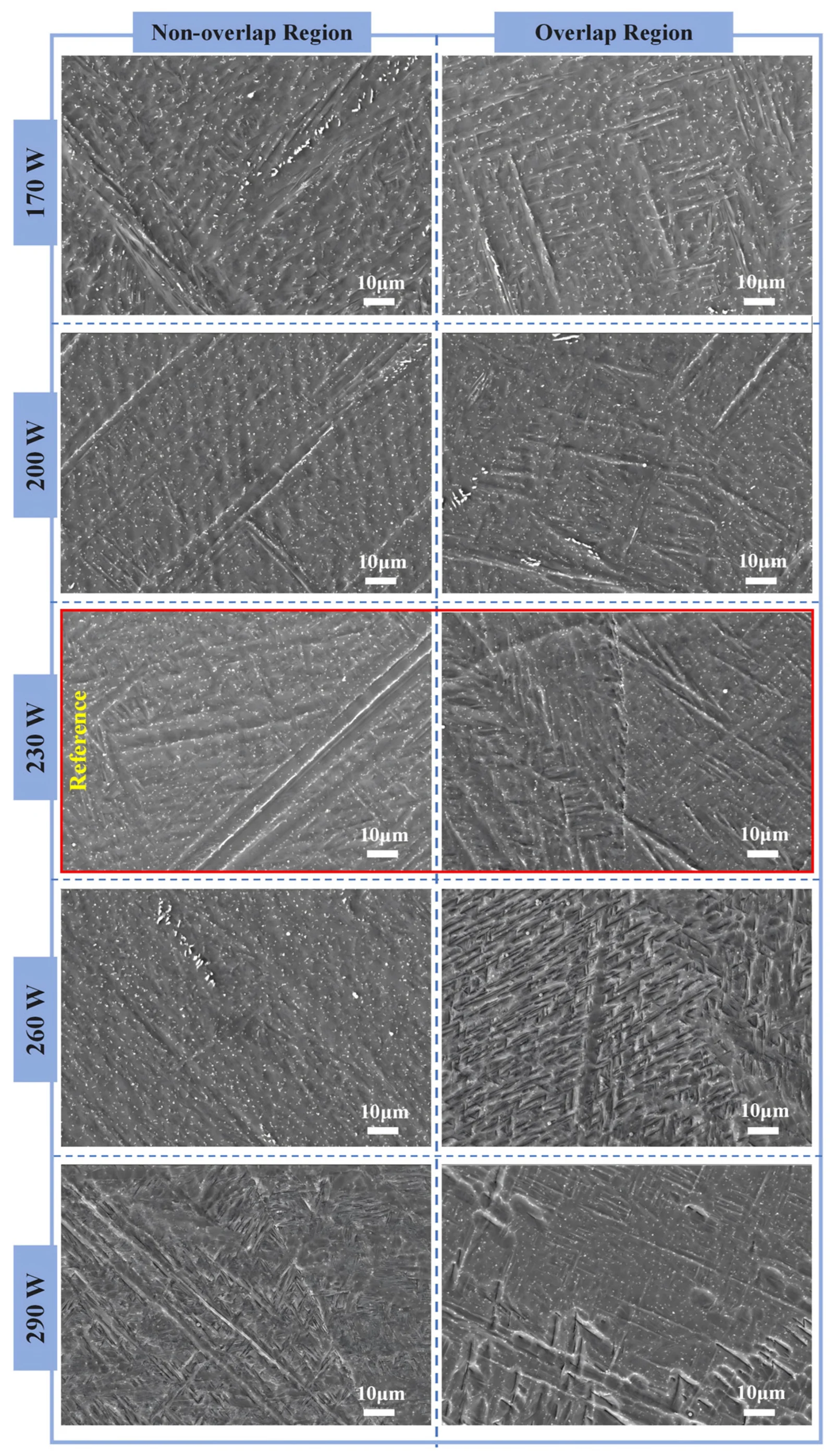
SEM microstructures of specimens fabricated with different laser powers. And the red box indicates the reference group.

The corresponding microstructures are presented in Figure 9. All specimens exhibited the characteristic acicular–lamellar morphology of PBF-ML TC11. Semi-quantitative measurements further showed a progressive increase in the apparent acicular-feature width with increasing laser power. As shown in Table 4, the measured width range increased from approximately 0.31−1.79 μm at 170 W to 0.75−3.15 μm at 290 W, while the reference condition at 230 W exhibited an intermediate range of 0.42−2.08 μm. This quantitative trend supports the SEM observation that increasing laser power promotes the development of relatively coarser and more continuous acicular–lamellar features. The increase in characteristic microstructural scale is consistent with the enhanced thermal exposure and repeated reheating associated with higher laser power.

The laser-power series indicates that the tensile response results from the coupled evolution of fusion quality, defect characteristics, and the thermally modified acicular–lamellar architecture. Increasing laser power within an appropriate range improves melting and load-bearing continuity, whereas excessive thermal exposure is accompanied by increased defect severity and measurable microstructural coarsening. The resulting tensile response therefore reflects the combined effects of fusion quality, defect characteristics, and microstructural scale rather than any single factor.

### 3.2. Effect of Scanning Speed on Defect–Microstructure Evolution and Tensile Response

**Figure 10:**
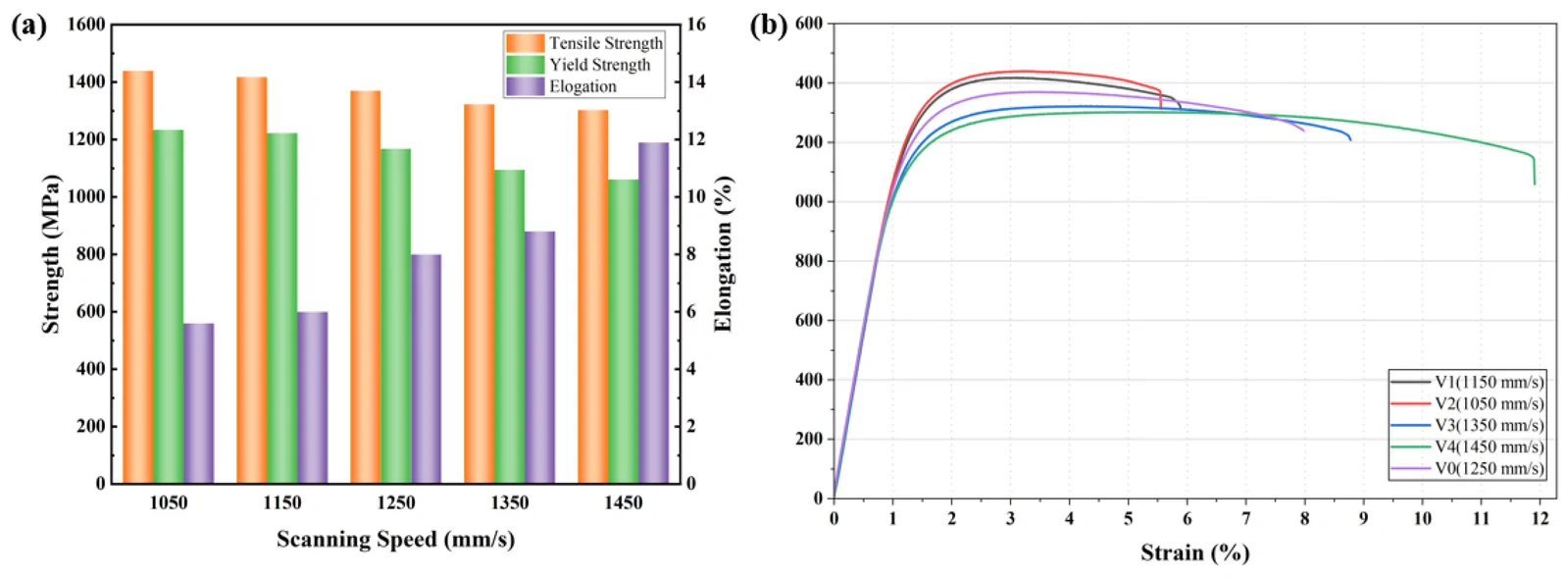
Tensile properties of specimens fabricated with different scanning speeds: (**a**) strength and elongation values and (**b**) engineering stress–strain curves.

The influence of scanning speed on the tensile response of PBF-ML-fabricated TC11 arch structures is shown in Figure 10. Increasing the scanning speed from 1050 to 1450 mm/s resulted in a gradual decrease in strength and an increase in ductility. The 0.2% proof strength (YS) decreased from 1234 to 1061 MPa, and the ultimate tensile strength (UTS) decreased from 1440 to 1303 MPa, whereas elongation (EL) increased from 5.6% to 11.9%. The engineering stress–strain curves exhibited similar elastic behavior, while the specimens fabricated at higher scanning speeds showed a longer plastic deformation stage before fracture. This indicates that scanning speed significantly influences the strength–ductility balance by modifying the laser–material interaction time and the associated thermal exposure during deposition.

At relatively low scanning speeds, the increased interaction time between the laser beam and powder bed leads to higher local thermal accumulation. The enhanced thermal exposure promotes sufficient melting and improves fusion continuity, contributing to higher strength. However, prolonged thermal cycling may also accelerate microstructural coarsening and increase the continuity of lamellar features, which can reduce the capacity for plastic deformation. In contrast, increasing the scanning speed reduces the energy delivered per unit length and shortens the melt-pool lifetime. Although excessive reduction in thermal input may increase the probability of incomplete fusion, the reduced thermal accumulation can preserve a finer and more dispersed acicular–lamellar structure, thereby improving strain accommodation and ductility.

**Figure 11:**
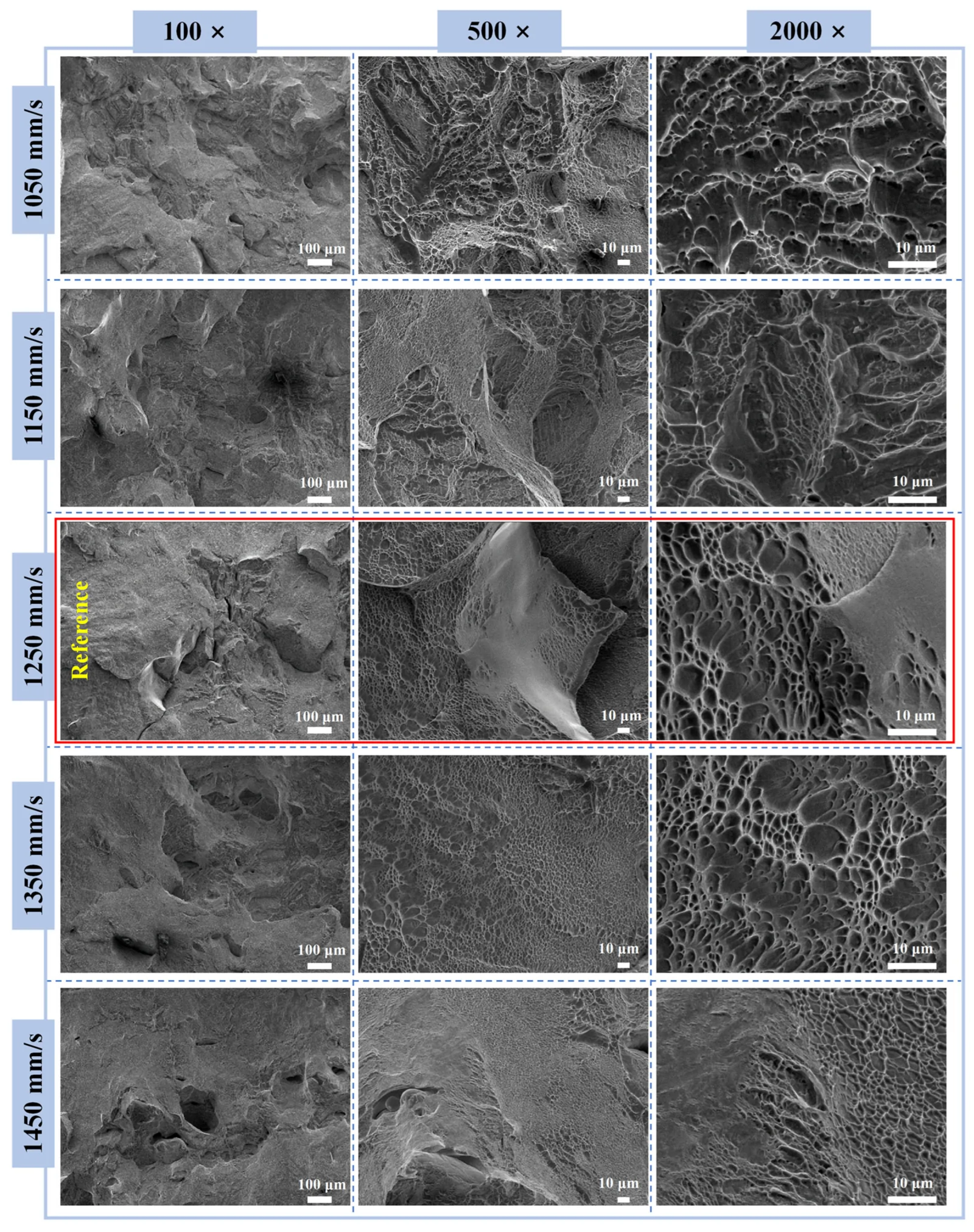
Fracture morphologies of specimens fabricated with different scanning speeds. The columns correspond to magnifications of 100×, 500×, and 2000× from left to right. And the red box indicates the reference group.

The fracture morphologies obtained under different scanning speeds are presented in Figure 11. All conditions exhibited predominantly ductile fracture characteristics. At 100× magnification, the low-speed specimens showed greater macroscopic fracture heterogeneity, whereas the fracture surfaces became more uniform with increasing scanning speed. At 500× magnification, localized cavity-like depressions and tearing features were more evident under the lower-speed conditions, indicating locally concentrated damage. At 2000× magnification, microvoid-coalescence features were observed, and the higher-speed specimens generally exhibited a finer and more uniformly distributed dimple morphology. These features are consistent with the increased elongation observed at higher scanning speeds.

**Figure 12:**
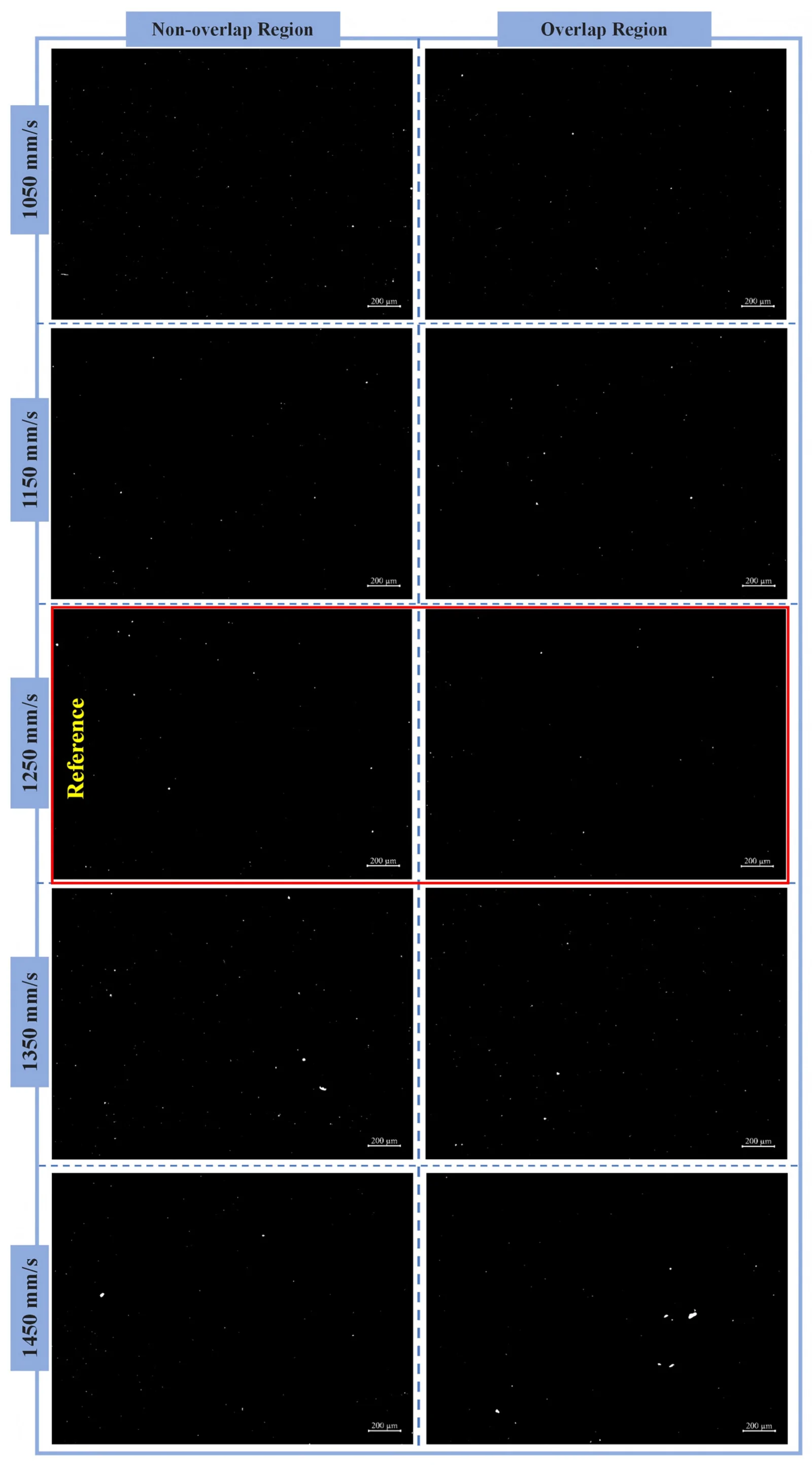
Binary defect maps obtained from polished cross-sections of specimens fabricated with different scanning speeds. And the red box indicates the reference group.

The binary defect maps obtained from polished cross-sections are shown in Figure 12, and the corresponding apparent two-dimensional defect statistics are summarized in Table 5. Neither defect count nor defect area fraction exhibited a simple monotonic relationship with scanning speed. The average defect counts were 339, 105, 91, 239, and 148 at scanning speeds of 1050, 1150, 1250, 1350, and 1450 mm/s, respectively, with the minimum count occurring at 1250 mm/s. The corresponding mean defect area fractions were approximately 0.153%, 0.125%, 0.122%, 0.172%, and 0.161%, respectively, with the largest value occurring at 1350 mm/s. The lack of synchronization between these two descriptors further demonstrates that defect count alone cannot fully characterize the defect state.

At low scanning speeds, increased thermal accumulation may promote melt-pool instability despite sufficient melting. At high scanning speeds, the reduced laser–material interaction time may compromise local fusion stability and increase the probability of incomplete-fusion-related defects. Therefore, the observed defect evolution reflects the competing effects of excessive thermal exposure and insufficient interaction time rather than a simple dependence on scanning speed.

The comparison between the inter-laser overlap zone and non-overlap region is also shown in Figure 12. The overlap zone generally exhibited a lower apparent defect count, whereas the defect area fraction showed a condition-dependent response. This difference indicates that the local effect of inter-laser interaction cannot be evaluated solely from defect frequency and should instead be interpreted by considering both defect number and projected area.

**Figure 13:**
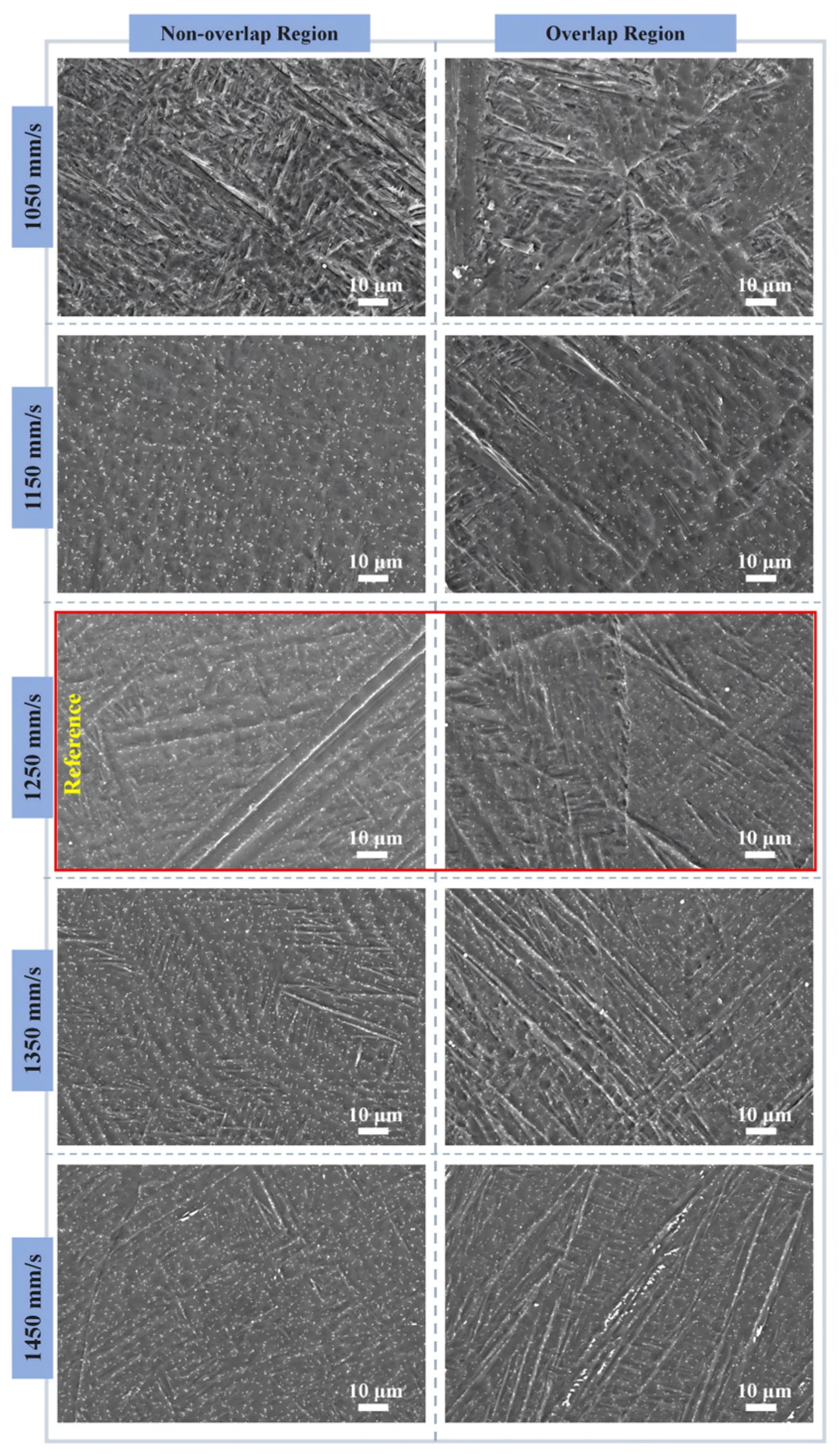
SEM microstructures of specimens fabricated with different scanning speeds. And the red box indicates the reference group.

The microstructures corresponding to different scanning speeds are presented in Figure 13. All specimens retained the characteristic acicular–lamellar morphology of PBF-ML TC11. Semi-quantitative measurements showed an overall reduction in apparent acicular-feature width with increasing scanning speed. As shown in Table 6, the measured width range decreased from approximately 0.68−3.45 μm at 1050 mm/s to 0.32−1.75 μm at 1450 mm/s, with the reference condition at 1250 mm/s exhibiting a range of 0.42–2.08 μm. Although some overlap existed among the measured ranges, the overall shift toward smaller characteristic widths supports the observed refinement of the acicular–lamellar structure at higher scanning speeds. This trend is consistent with the reduced laser–material interaction time, thermal accumulation, and repeated reheating at higher scanning speeds.

The scanning-speed results indicate that the tensile response is governed by the coupled effects of melt-pool/fusion stability, critical-defect characteristics, and thermally induced changes in the acicular–lamellar structure. Lower scanning speeds increased laser–material interaction time and thermal exposure, promoting sufficient melting but also coarser acicular–lamellar features. Increasing scanning speed reduced the characteristic microstructural scale and improved ductility, although excessive reductions in interaction time may compromise local fusion stability. The resulting strength–ductility response therefore reflects competition among fusion quality, defect characteristics, and microstructural evolution.

### 3.3. Influence of Overlap-Zone Position on Local Heterogeneity and Tensile Response

**Figure 14:**
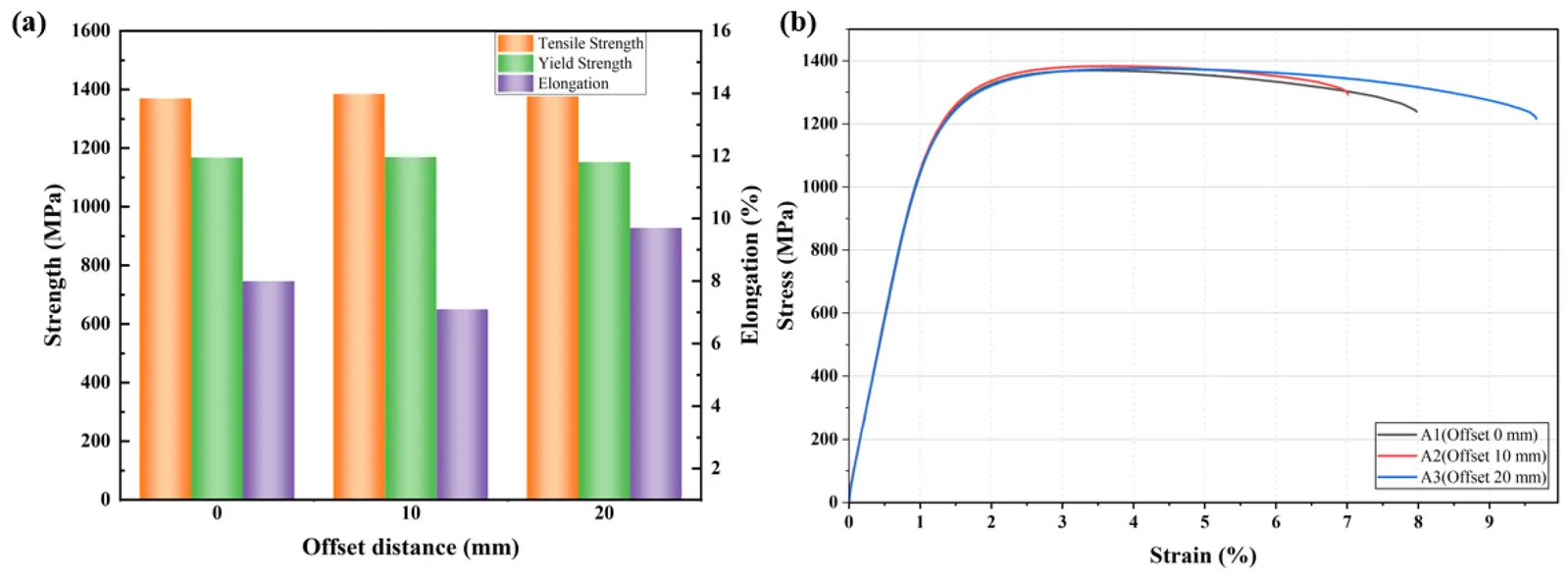
Tensile properties of specimens with different overlap-zone positions: (**a**) strength and elongation values and (**b**) engineering stress–strain curves.

The influence of overlap-zone position on the tensile response of PBF-ML-fabricated TC11 arch structures is shown in Figure 14. Unlike the laser-power and scanning-speed series, the overlap-zone-position series was performed under identical laser power and scanning speed conditions, allowing the effect of the spatial location of the inter-laser overlap zone relative to the arch geometry to be evaluated independently.

The engineering stress–strain curves of the three overlap-zone positions exhibited similar deformation behavior, indicating comparable tensile responses among the investigated conditions. The YS varied from 1153 to 1170 MPa, while the UTS ranged from 1370 to 1389 MPa. The elongation showed relatively larger variation, ranging from 7.1% to 9.7%. Compared with the variations induced by laser power and scanning speed, the influence of overlap-zone position on the overall tensile strength was limited.

This behavior can be attributed to the fact that relocating the overlap zone does not directly change the nominal thermal input delivered during fabrication. Instead, it modifies the spatial relationship between the laser-field boundary and the geometrically constrained arch structure, thereby affecting the local distribution of defects and microstructural heterogeneity. Since the overall melting conditions and thermal input remained unchanged, the matrix microstructure and intrinsic load-bearing capability were generally maintained. Therefore, the overlap-zone position mainly influenced local deformation behavior rather than the overall strength level.

**Figure 15:**
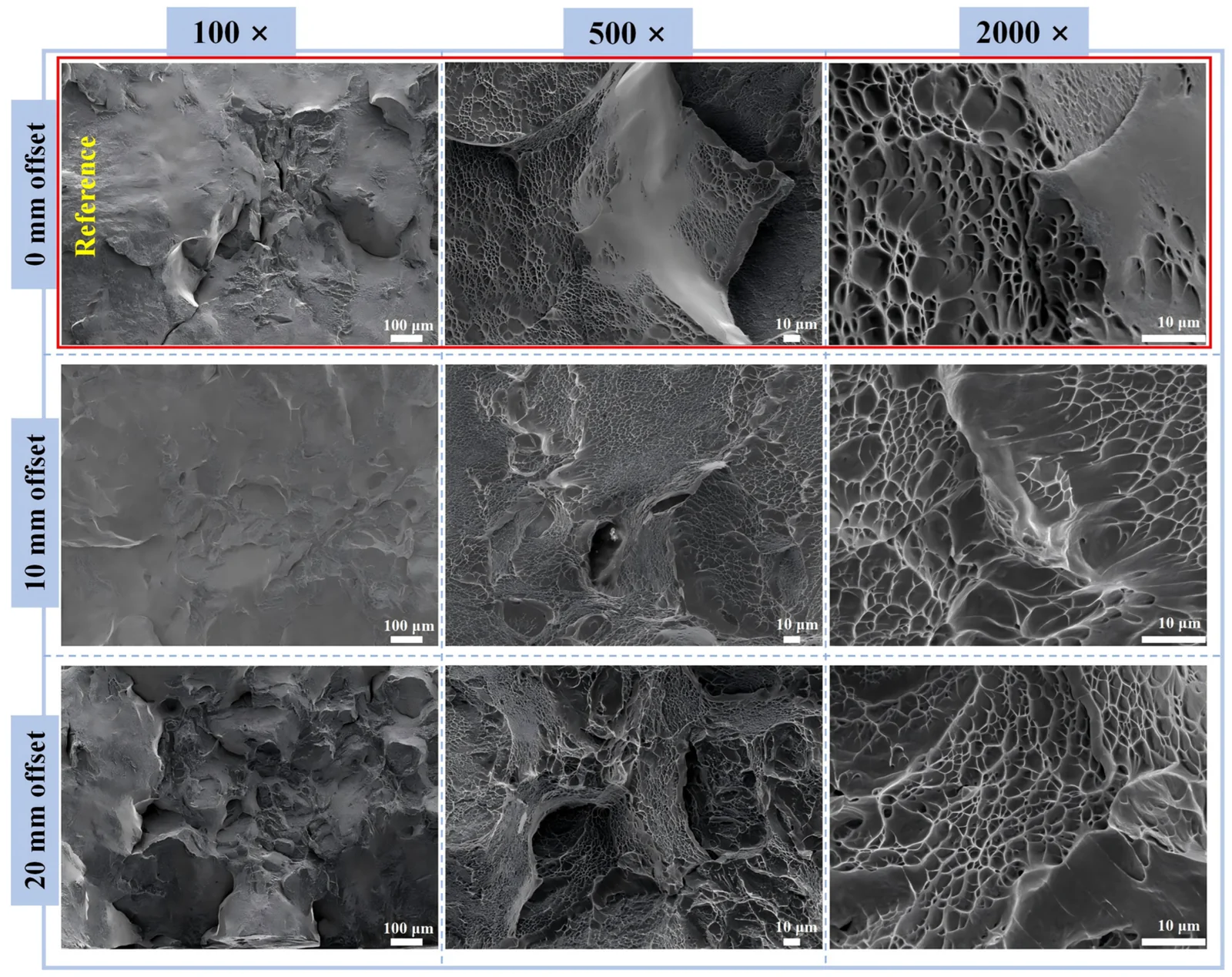
Fracture morphologies of specimens with different overlap-zone positions. The columns correspond to magnifications of 100×, 500×, and 2000× from left to right. And the red box indicates the reference group.

The fracture morphologies corresponding to different overlap-zone positions are presented in Figure 15. All conditions exhibited predominantly ductile fracture characteristics, with no apparent transition in the overall fracture mode. At 100× magnification, the three conditions showed generally similar macroscopic fracture characteristics, although localized heterogeneous regions were more evident for A2. At 500× magnification, A2 exhibited more pronounced localized cavity-like features, whereas A3 showed stronger tearing characteristics. At 2000× magnification, A1 exhibited a relatively uniform distribution of fine dimples, while A3 showed relatively deeper and more developed dimples. These local differences are consistent with the observed variations in elongation and suggest differences in local damage accumulation rather than a systematic change in the overall fracture mechanism.

**Figure 16:**
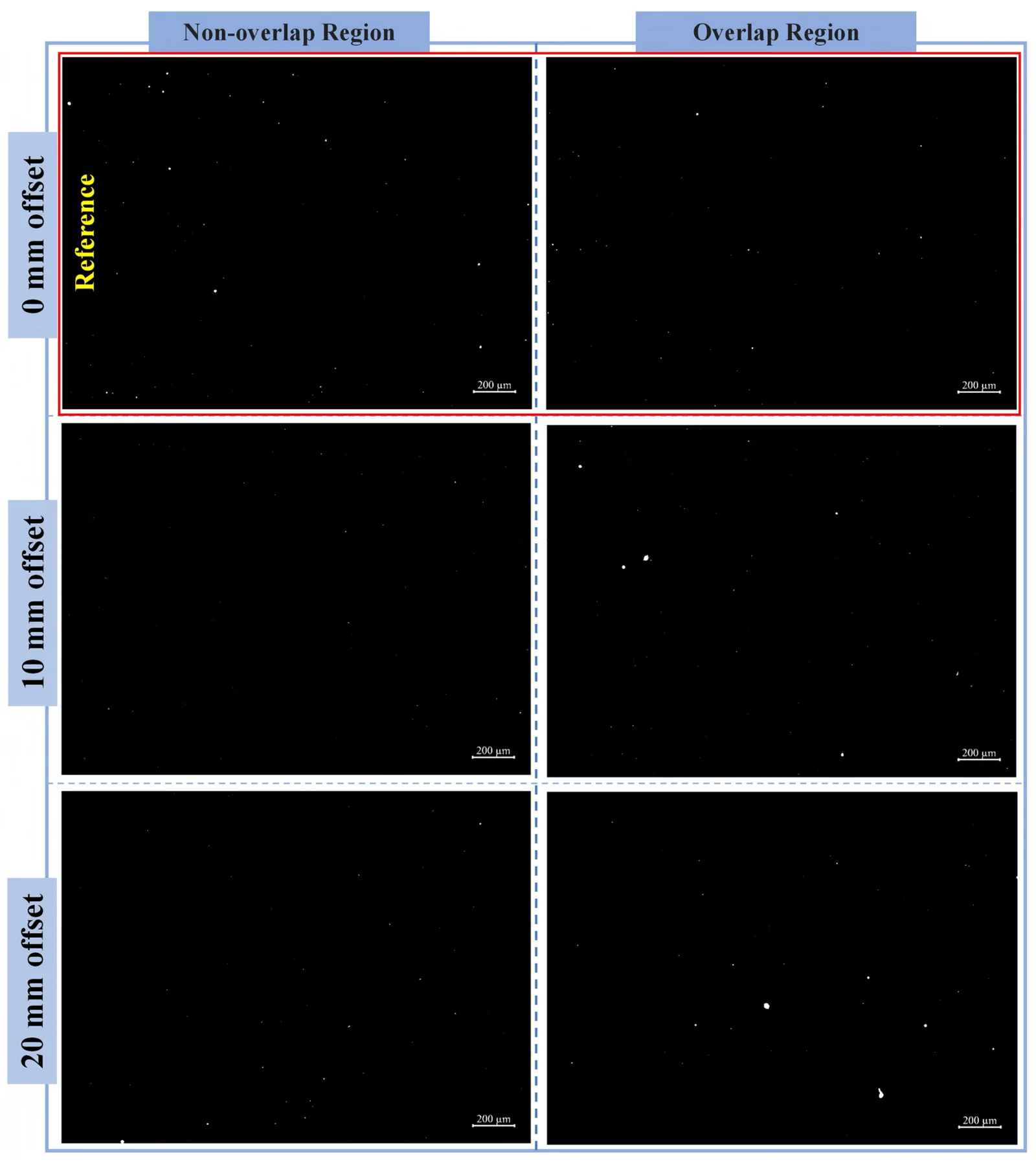
Binary defect maps obtained from polished cross-sections of specimens with different overlap-zone positions. And the red box indicates the reference group.

The binary defect maps for different overlap-zone positions are shown in Figure 16, and the corresponding apparent two-dimensional defect frequencies are summarized in Table 7. The average detected defect counts were 91, 82, and 53 for A1, A2, and A3, respectively. Although the average defect count decreased from 91 for A1 to 53 for A3, the corresponding mean defect area fractions remained comparable at approximately 0.122%, 0.115%, and 0.123% for A1, A2, and A3, respectively. Therefore, the reduction in the number of detected defects at the 20 mm offset did not correspond to a comparable reduction in the total projected defect area. This result indicates that changes in defect number were accompanied by changes in the characteristic defect size and further explains why defect count alone did not correlate directly with the tensile response.

Within the examined two-dimensional fields, the overlap region consistently exhibited a lower defect count than the corresponding non-overlap region. However, the defect area fraction did not decrease consistently, particularly for the 20 mm offset condition. These results indicate that relocating the overlap zone mainly changed the number, characteristic size, and spatial distribution of detectable defects rather than producing a uniform reduction in the overall defect content.

**Figure 17:**
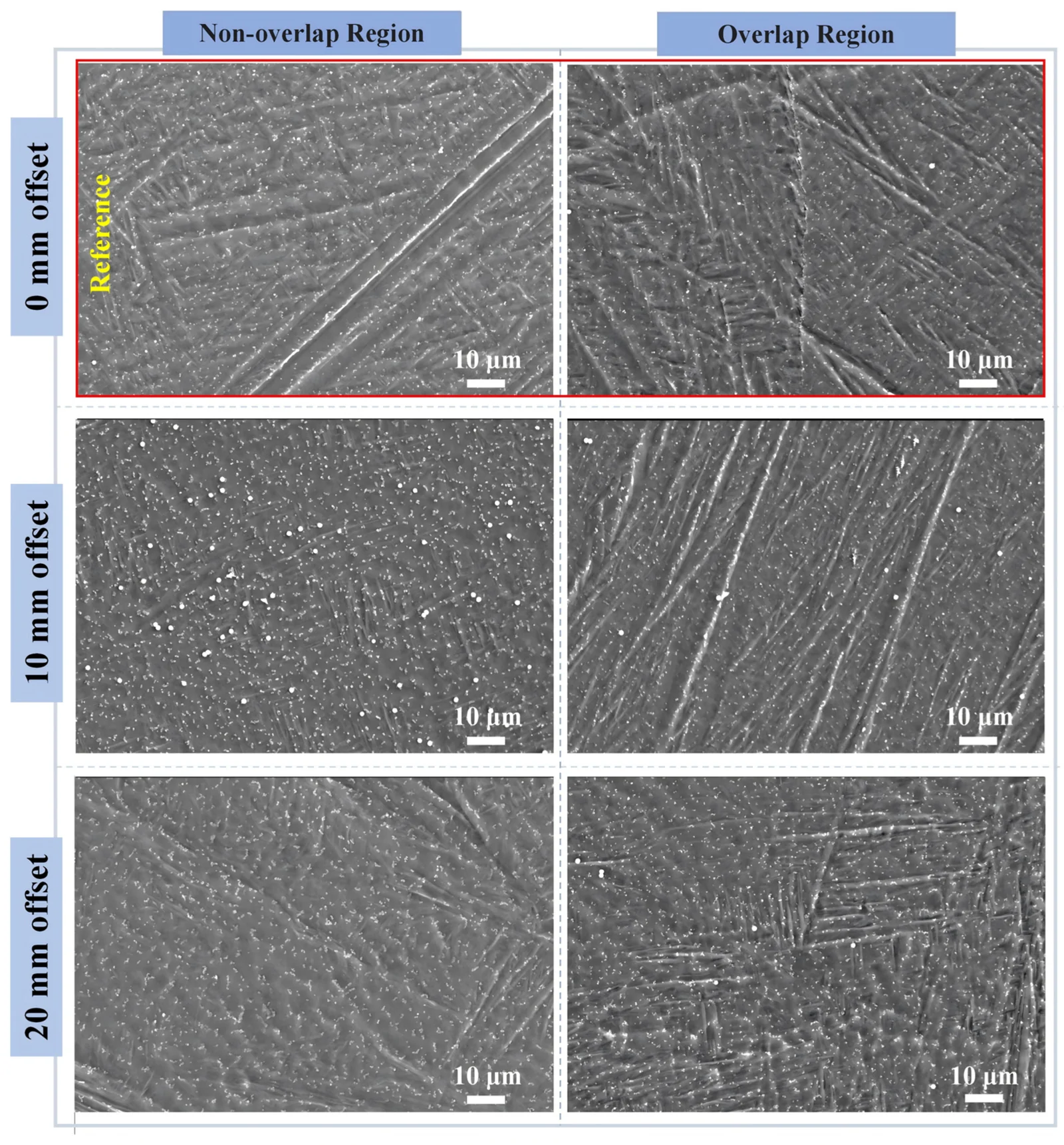
SEM microstructures associated with different overlap-zone positions. And the red box indicates the reference group.

The microstructural characteristics associated with different overlap-zone positions are shown in Figure 17. All conditions exhibited broadly similar acicular–lamellar morphologies, indicating that relocating the overlap zone did not produce a pronounced change in the overall microstructural morphology observable by SEM. However, local differences in packet arrangement and directional continuity of the observable lamellar features were evident between the sampled regions. At the 10 mm offset, more pronounced local differences in lamellar arrangement were observed, whereas the differences at the 20 mm offset were less systematic. Overall, these observations indicate that overlap-zone position modifies local microstructural heterogeneity without causing a pronounced change in the global acicular–lamellar morphology.

The overlap-zone-position results therefore indicate that relocating the inter-laser boundary primarily modifies local defect and microstructural heterogeneity. Because the nominal laser power and scanning speed remained unchanged, the limited variations in tensile strength are consistent with largely preserved overall melting conditions, while local differences in defect distribution and microstructural arrangement contribute mainly to variations in deformation behavior.

### 3.4. Influence of Interlayer Rotation on Thermal-Path Distribution and Deformation Response

**Figure 18:**
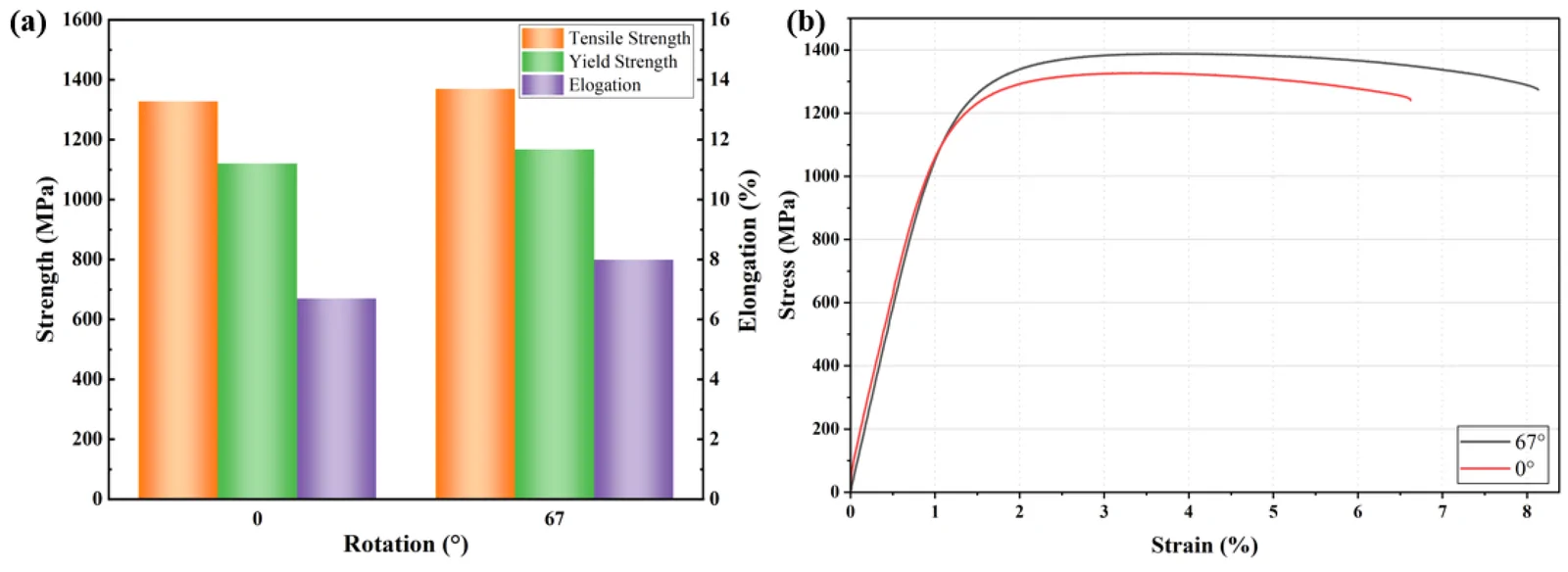
Tensile properties of specimens fabricated with different interlayer rotation strategies: (**a**) strength and elongation values and (**b**) engineering stress–strain curves.

The influence of interlayer-rotation strategy on the tensile response of multi-laser fabricated TC11 arch structures is shown in Figure 18. Compared with repeated scanning without interlayer rotation (R0), the 67° rotation strategy (R67) increased the YS from 1121 to 1168 MPa, the UTS from 1328 to 1370 MPa, and the EL from 6.7% to 8.0%. The engineering stress–strain curves exhibited similar elastic deformation behavior, whereas the R67 condition maintained a slightly higher flow stress and a longer plastic deformation stage before fracture.

Unlike laser power and scanning speed, interlayer rotation does not directly modify the nominal energy input. Instead, its influence mainly arises from changes in the spatial arrangement of scan vectors and the resulting layer-wise thermal cycling paths. Repeated scanning without rotation maintains similar heat-flow directions between successive layers, which may promote directional thermal accumulation and localized deformation. In contrast, the 67° rotation strategy continuously changes the orientation of scanning trajectories, reducing the persistence of identical thermal paths and modifying the spatial distribution of thermal cycling and potentially altering the associated local stress development. Therefore, the moderate improvement in both strength and elongation under the rotated condition is consistent with a more spatially distributed deformation response rather than a substantial change in the nominal thermal input.

**Figure 19:**
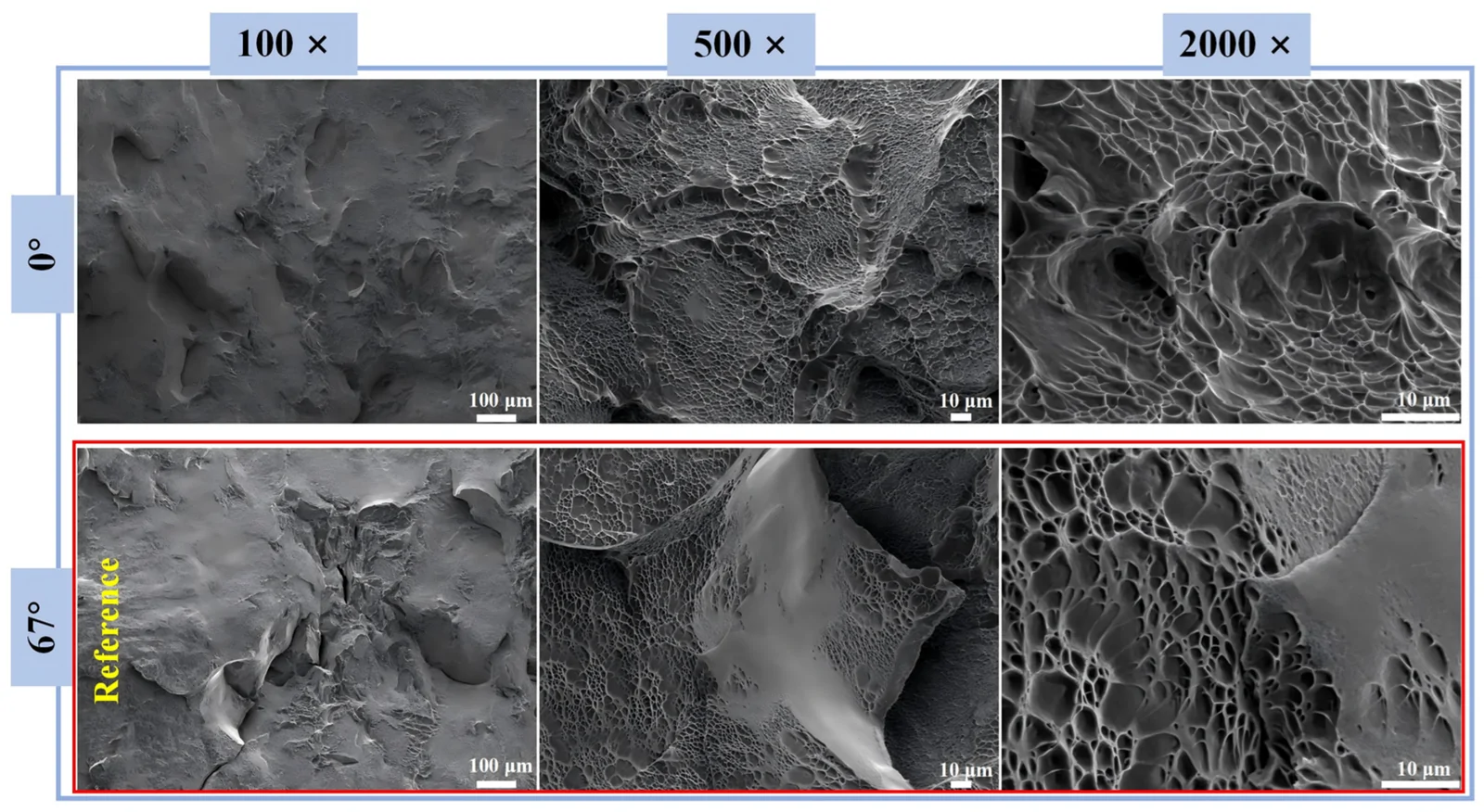
Fracture morphologies of specimens fabricated with different interlayer-rotation strategies. The columns correspond to magnifications of 100×, 500×, and 2000× from left to right. And the red box indicates the reference group.

The fracture morphologies of the two scanning strategies are compared in Figure 19. Both conditions exhibited typical ductile fracture characteristics dominated by microvoid coalescence, indicating no fundamental change in fracture mode. At 100× magnification, the R67 condition exhibited a relatively rougher and more heterogeneous fracture surface, whereas the R0 condition showed comparatively flatter regions. At 500× magnification, R67 exhibited more tortuous tearing features, while more localized cavity coalescence was observed for R0. At 2000× magnification, both conditions showed dimpled fracture characteristics, with R67 exhibiting a more distributed dimple morphology. These observations suggest a more spatially distributed deformation response under the 67° rotation condition, consistent with its moderately higher strength and elongation.

**Figure 20:**
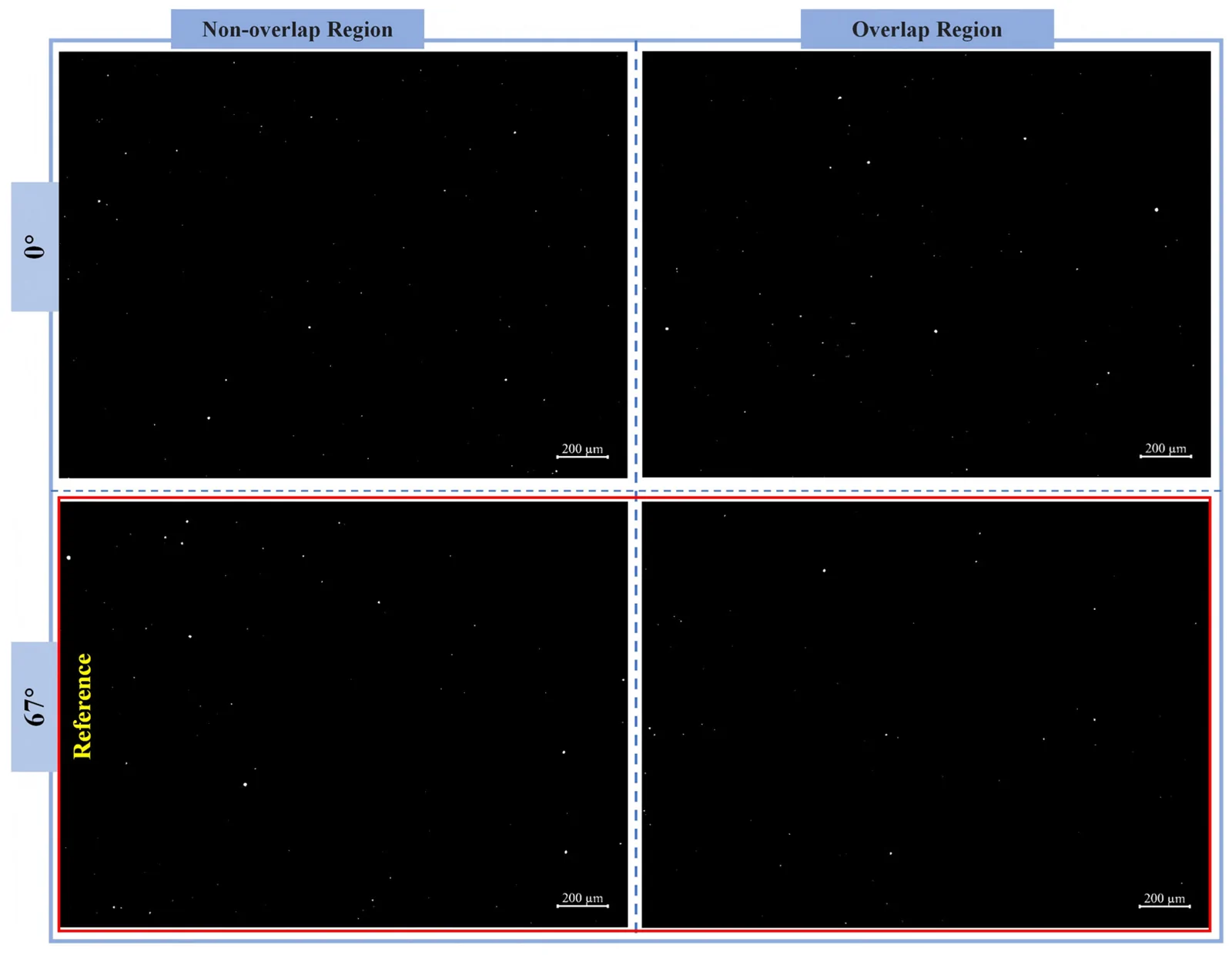
Binary defect maps obtained from polished cross-sections of specimens fabricated with different interlayer-rotation strategies. And the red box indicates the reference group.

The binary defect maps for the interlayer-rotation conditions are presented in Figure 20, and the corresponding apparent two-dimensional defect frequencies are summarized in Table 8. Although the average defect count decreased from 126 for R0 to 91 for R67, the corresponding mean defect area fraction changed only slightly, from approximately 0.126% to 0.122%. This comparison indicates that interlayer rotation primarily altered the number and distribution of detectable defect features rather than producing a substantial reduction in the total projected defect area.

The lower defect count under the R67 condition suggests that changing the scan-vector orientation modified the spatial distribution of detectable defects. Because laser power and scanning speed remained unchanged, this difference is more reasonably associated with the redistribution of layer-wise thermal cycling than with a change in nominal energy input. Meanwhile, the overlap region exhibited a lower apparent defect count under both rotation strategies, whereas the corresponding defect area fractions showed only small and condition-dependent differences. These results indicate that interlayer rotation and inter-laser interaction primarily modified the number, size-related characteristics, and spatial distribution of detectable defects rather than simply reducing the overall defect content.

**Figure 21:**
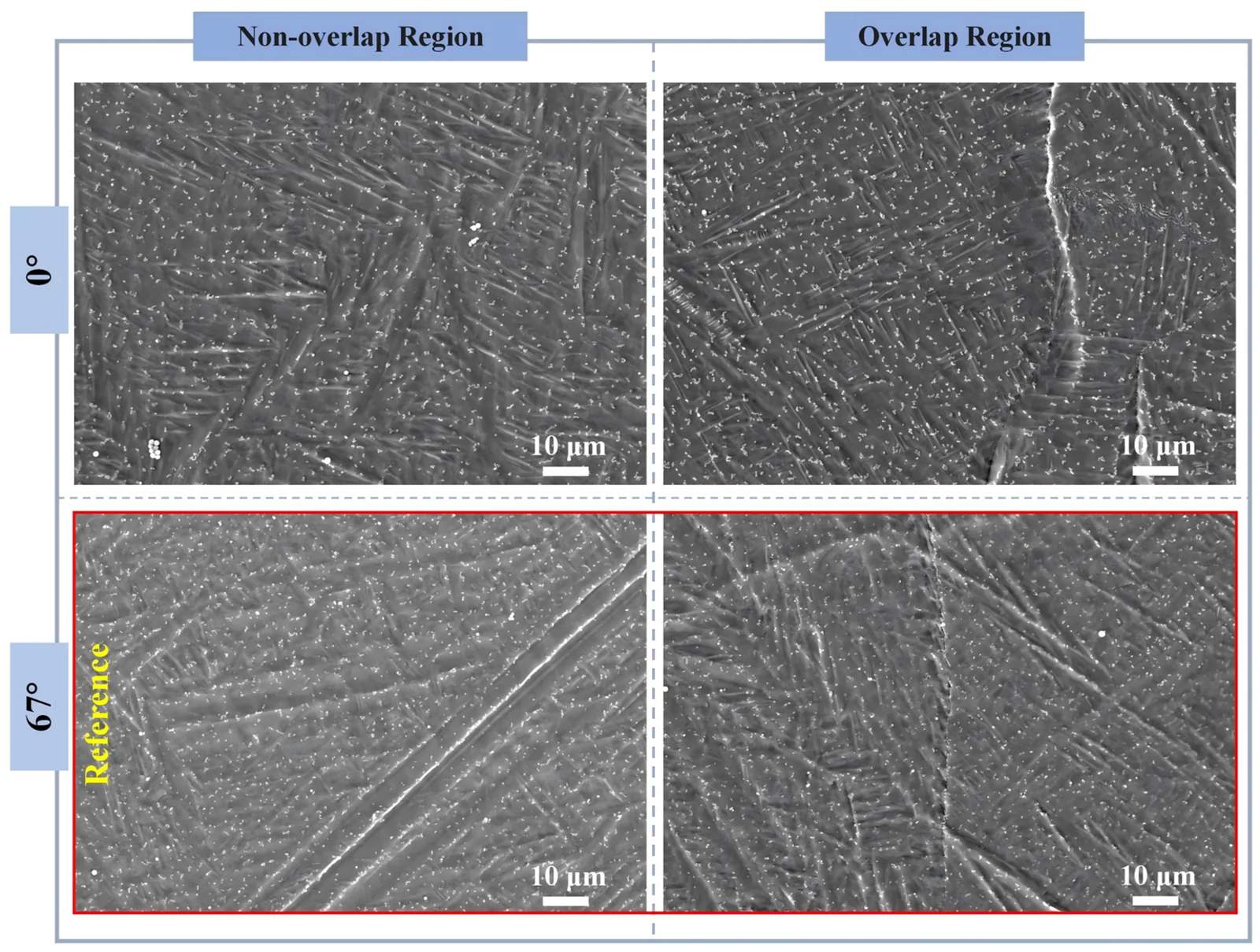
SEM microstructures of the inter-laser overlap zone and non-overlap region fabricated with different interlayer-rotation strategies. And the red box indicates the reference group.

The corresponding microstructures are shown in Figure 21. Both scanning strategies exhibited broadly similar acicular–lamellar morphologies, with no pronounced difference in the overall microstructural features observable by SEM. However, differences in packet arrangement and the directional distribution of the observable lamellar features were evident. The R67 condition exhibited a more dispersed and interwoven packet morphology, whereas the R0 condition showed a stronger tendency toward directional continuity in some regions. These differences suggest that interlayer rotation mainly modified the spatial arrangement of the acicular–lamellar microstructure rather than producing a major change in its overall morphology.

The interlayer-rotation results indicate that changing the scan-vector orientation between layers provides an effective approach for modifying thermal-path directionality under constant processing conditions. The 67° rotation strategy was associated with a lower apparent defect frequency, a more dispersed packet arrangement, and a more tortuous ductile fracture morphology. The modest improvements in YS, UTS, and EL are therefore interpreted as the combined result of changes in defect distribution, local microstructural arrangement, and deformation uniformity induced by the redistributed layer-wise thermal paths rather than as the consequence of a single microstructural mechanism.

## 4. Conclusions

This study investigated the effects of laser power, scanning speed, overlap-zone position, and interlayer rotation on the tensile properties, apparent defect frequency, fracture morphology, and acicular–lamellar microstructure of PBF-ML-fabricated TC11 arch structures. The main conclusions are as follows:

(1) Increasing the laser power from 170 to 290 W increased YS from 955 to 1259 MPa and UTS from 1228 to 1463 MPa, while EL decreased from 14.7% to 3.1%. The average apparent defect count first decreased from 229 at 170 W to a minimum of 91 at 230 W and then increased to 383 at 290 W. Higher-power conditions exhibited progressively coarser acicular–lamellar features, with the apparent acicular-feature width increasing from approximately 0.31–1.79 μm at 170 W to 0.75–3.15 μm at 290 W, corresponding to the pronounced loss in ductility. 

(2) Increasing the scanning speed from 1050 to 1450 mm/s decreased YS from 1234 to 1061 MPa and UTS from 1440 to 1303 MPa, while EL increased from 5.6% to 11.9%. The corresponding average apparent defect counts were 339, 105, 91, 239, and 148. With increasing scanning speed, the apparent acicular-feature width decreased overall from approximately 0.68–3.45 μm at 1050 mm/s to 0.32–1.75 μm at 1450 mm/s, supporting the observed tendency toward a finer acicular–lamellar structure and improved ductility. 

(3) Changing the overlap-zone position produced only limited variations in strength, with YS ranging from 1153 to 1170 MPa and UTS ranging from 1370 to 1389 MPa, while EL ranged from 7.1% to 9.7%. Although the average defect count decreased from 91 to 53 as the offset increased from 0 to 20 mm, the corresponding mean defect area fraction remained nearly unchanged. The overall acicular–lamellar morphology was also similar among the three conditions, indicating that overlap-zone position primarily modified local defects and microstructural heterogeneity rather than the overall material response.

(4) Compared with repeated scanning without interlayer rotation, the 67° rotation condition increased YS from 1121 to 1168 MPa, UTS from 1328 to 1370 MPa, and EL from 6.7% to 8.0%, while the average defect count decreased from 126 to 91. The corresponding defect area fraction changed only slightly, and both conditions exhibited similar overall acicular–lamellar morphologies. The principal differences were associated with local packet arrangement and fracture morphology, indicating that interlayer rotation mainly modified local heterogeneity rather than the overall microstructural scale.

Overall, laser power and scanning speed produced the most pronounced changes in tensile response, defect characteristics, and microstructural scale, whereas overlap-zone position and interlayer rotation mainly modified local heterogeneity. Defect count and area fraction did not always vary synchronously, demonstrating that defect frequency alone is insufficient to characterize the mechanical response. Defect-size-related characteristics, microstructural scale, and fracture behavior should therefore be considered together when evaluating PBF-ML TC11 structures.

## Figures and Tables

**Table 1:** Nominal chemical composition of the TC11 titanium-alloy powder.

Element	Ti	Al	Mo	Zr	Si	Fe	C	O	N	H
wt.%	Bal.	6.860	3.570	1.730	0.310	0.033	0.012	0.120	0.006	0.003

**Table 2:** Processing variables and levels investigated in the PBF-ML experiments.

Series	Specimen	Level	Unit	Status
Laser power	P2	170	W	—
	P1	200		—
	P0	230		Reference
	P3	260		—
	P4	290		—
Scan speed	V2	1050	mm/s	—
	V1	1150		—
	V0 (P0)	1250		Reference
	V3	1350		—
	V4	1450		—
Overlap offset	A1 (P0)	0	mm	Reference
	A2	10		—
	A3	20		—
Rotation angle	R0	0	°	—
	R67 (P0)	67		Reference

**Table 3:** Apparent two-dimensional defect statistics for specimens fabricated with different laser powers.

ID	Process Level	Non-Overlap Region Count	Overlap Region Count	Average Count	Non-Overlap Defect Area Fraction (%)	Overlap Region Defect Area Fraction (%)
P2	170 W	236	222	229	0.4824	0.5174
P1	200 W	113	97	105	0.1164	0.1045
P0	230 W	116	66	91	0.1336	0.1100
P3	260 W	224	158	196	0.1556	0.1432
P4	290 W	458	308	383	0.2483	0.2033

**Table 4:** Semi-quantitative apparent acicular-feature width under different laser-power conditions.

ID	Process Level	Approximate Apparent Acicular-Feature Width (μm)
P2	170 W	0.31−1.79
P1	200 W	0.39−1.89
P0	230 W	0.42−2.08
P3	260 W	0.66−2.62
P4	290 W	0.75−3.15

**Table 5:** Apparent two-dimensional defect statistics for specimens fabricated with different scanning speeds.

ID	Scanning Speed	Non-Overlap Region Count	Overlap Region Count	Average Count	Non-Overlap Defect Area Fraction (%)	Overlap Region Defect Area Fraction (%)
V2	1050 mm/s	402	276	339	0.1647	0.1405
V1	1150 mm/s	106	104	105	0.1236	0.1269
V0	1250 mm/s	116	66	91	0.1336	0.1100
V3	1350 mm/s	245	233	239	0.1887	0.1548
V4	1450 mm/s	184	112	148	0.1384	0.1829

**Table 6:** Semi-quantitative apparent acicular-feature width under different scanning-speed conditions.

ID	Process Level	Approximate Apparent Acicular-Feature Width (μm)
V2	1050 mm/s	0.68–3.45
V1	1150 mm/s	0.38–2.46
V0	1250 mm/s	0.42–2.08
V3	1350 mm/s	0.37–1.93
V4	1450 mm/s	0.32–1.75

**Table 7:** Apparent two-dimensional defect statistics for specimens with different overlap-zone positions.

ID	Offset	Non-Overlap Region	Overlap Region	Average Count	Non-Overlap Defect Area Fraction (%)	Overlap Region Defect Area Fraction (%)
A1	0 mm	116	66	91	0.1336	0.1100
A2	10 mm	102	62	82	0.1329	0.0961
A3	20 mm	56	50	53	0.1072	0.1386

**Table 8:** Apparent two-dimensional defect statistics for specimens fabricated with different interlayer-rotation strategies.

ID	Rotation	Non-Overlap Region	Overlap Region	Average Count	Non-Overlap Defect Area Fraction (%)	Overlap Region Defect Area Fraction (%)
R0	0°	135	117	126	0.1193	0.1327
R67	67°	116	66	91	0.1336	0.1100

## Data Availability

The original contributions presented in this study are included in the article. Further inquiries can be directed to the corresponding author.
